# Diminished neutrophil extracellular trap (NET) formation is a novel innate immune deficiency induced by acute ethanol exposure in polymicrobial sepsis, which can be rescued by CXCL1

**DOI:** 10.1371/journal.ppat.1006637

**Published:** 2017-09-18

**Authors:** Liliang Jin, Sanjay Batra, Samithamby Jeyaseelan

**Affiliations:** 1 Laboratory of Lung Biology, Department of Pathobiological Sciences and Center for Experimental Infectious Disease Research, School of Veterinary Medicine, Louisiana State University, Baton Rouge, Louisiana, United States of America; 2 Division of Pulmonary and Critical Care, Department of Medicine, LSU Health Sciences Center, New Orleans, Louisiana, United States of America; University of California Davis School of Medicine, UNITED STATES

## Abstract

Polymicrobial sepsis is the result of an exaggerated host immune response to bacterial pathogens. Animal models and human studies demonstrate that alcohol intoxication is a key risk factor for sepsis-induced mortality. Multiple chemokines, such as CXCL1, CXCL2 and CXCL5 are critical for neutrophil recruitment and proper function of neutrophils. However, it is not quite clear the mechanisms by which acute alcohol suppresses immune responses and whether alcohol-induced immunosuppression can be rescued by chemokines. Thus, we assessed whether acute ethanol challenge via gavage diminishes antibacterial host defense in a sepsis model using cecal ligation and puncture (CLP) and whether this immunosuppression can be rescued by exogenous CXCL1. We found acute alcohol intoxication augments mortality and enhances bacterial growth in mice following CLP. Ethanol exposure impairs critical antibacterial functions of mouse and human neutrophils including reactive oxygen species production, neutrophil extracellular trap (NET) formation, and NET-mediated killing in response to both Gram-negative (*E*. *coli*) and Gram-positive (*Staphylococcus aureus)* pathogens. As compared with WT (C57Bl/6) mice, CXCL1 knockout mice display early mortality following acute alcohol exposure followed by CLP. Recombinant CXCL1 (rCXCL1) in acute alcohol challenged CLP mice increases survival, enhances bacterial clearance, improves neutrophil recruitment, and enhances NET formation (NETosis). Recombinant CXCL1 (rCXCL1) administration also augments bacterial killing by alcohol-treated and *E*. *coli-* and *S*. *aureus*-infected neutrophils. Taken together, our data unveils novel mechanisms underlying acute alcohol-induced dysregulation of the immune responses in polymicrobial sepsis, and CXCL1 is a critical mediator to rescue alcohol-induced immune dysregulation in polymicrobial sepsis.

## Author summary

Sepsis is still a leading cause of morbidity and mortality in critically ill patients. Multiple organ failure and mortality in sepsis is caused by uncontrolled activation of the immune system. This results in impaired ability to control bacterial colonization and dissemination along with excessive inflammation-induced pathology. Neutrophils are critical innate immune cells that provide the first line of defense against sepsis through their ability to rapidly migrate to the site of infection and restrict bacterial multiplication and dissemination. Alcohol intoxication is a key risk factor for sepsis-induced mortality. However, the mechanisms by which acute alcohol suppresses immune responses in sepsis and whether alcohol-induced immunosuppression in sepsis can be rescued by chemokines remain elusive. We found that acute alcohol intoxication augments mortality and enhances bacterial growth in septic mice. Alcohol exposure also impairs critical antibacterial functions of mouse and human neutrophils. Recombinant neutrophil chemokine (CXCL1) in acute alcohol challenged septic mice increases neutrophil-dependent host protection. Therefore, our study provides novel mechanisms underlying acute alcohol-induced dysregulation of the immune responses in sepsis which can be rescued by CXCL1.

## Introduction

Sepsis is a complex clinical manifestation of dysregulated host inflammatory responses to infection causing damage to vital organs that often results in death [1]. Treatment of sepsis requires prolonged intensive care and thus, results in an increased economic burden. It is well known that much of the pathophysiology of sepsis is the result of the excessive host response to bacterial pathogens [1]. An improved understanding of the initiation of the immune response to bacterial invasion is prerequisite to the designing of successful therapeutic strategies in order to limit off-target inflammation while still successfully controlling the bacterial burden in sepsis.

Clinical studies have shown that alcoholics are more susceptible to sepsis and thus have a higher mortality rate compared to non-alcoholics with sepsis [2]. Moreover, alcohol abuse is a leading risk factor for mortality, causing around 100,000 deaths per year [2, 3]. In addition, ethanol consumption is associated with an increased incidence and severity of a broad spectrum of natural infections in humans and experimental animals [1, 3]. Human studies and animal models unequivocally demonstrate that acute alcohol intoxication is a leading risk factor for mortality associated with sepsis [4–7]. However, the molecular and cellular pathways underlying this association are unclear.

While the innate immune response is essential for the restraint of bacterial growth, it is the uncontrolled innate response that contributes to host pathology during sepsis [1]. In particular, neutrophils are pivotal innate immune cells that provide the first line of defense against sepsis via their ability to rapidly migrate to the infectious focus (peritoneum) and restrict bacterial multiplication and dissemination to distant organs [8, 9]. Moreover, impaired neutrophil migration has been associated with increased mortality and higher bacterial burdens in human sepsis and in experimental models of sepsis [8, 10]. In our previous studies, the chemokine CXCL1 (aka KC) induces not only neutrophil recruitment to tissues but also neutrophil extracellular trap (NET)-mediated bacterial killing [11]

The formation of neutrophil extracellular traps (NETs), a process also known as NETosis, by polymorphonuclear leukocytes (PMNs) has been described as a novel function of neutrophils [12]. NETs are extracellular lattices of decondensed chromatin that contain antimicrobial proteases [12]. Emerging data show that NETs trap microbes, including bacteria, fungi, and some parasites [12, 13], and additional investigations have demonstrated that NETs kill extracellular microbes thereby limiting the spread of pathogens [12–14].

Alcohol is known to regulate phagocytosis, antigen presentation, and the production of antimicrobial molecules by monocytes, macrophages, and dendritic cells [15]. One of the prominent presenting features of alcohol abusers with sepsis or septicemia is reduced granulocyte recruitment at infectious focus and systemic granulocytopenia [16]. Nevertheless, the effects of alcohol on neutrophil antibacterial function, including NET formation (NETosis), during infection remain elusive.

In this study, we used a mouse model of polymicrobial sepsis via cecal-ligation and puncture (CLP) along with experiments conducted in primary mouse and human neutrophils to investigate the role of acute alcohol on innate defense mechanisms during sepsis. In addition, we have used CXCL1 knockout mice and recombinant CXCL1 (rCXCL1) to demonstrate whether neutrophil accumulation and function can be rescued in alcohol-challenged CLP mice. Our findings indicate that acute alcohol-mediated reductions in NETosis and NET-mediated extracellular killing of bacteria contribute to the impaired bacterial clearance in polymicrobial sepsis as a novel mechanism, and CXCL1 rescues antibacterial defense, such as neutrophil recruitment and function in alcohol-challenged CLP mice.

## Results

### Acute alcohol intoxicated mice are more susceptible to polymicrobial sepsis

**Fig 1:**
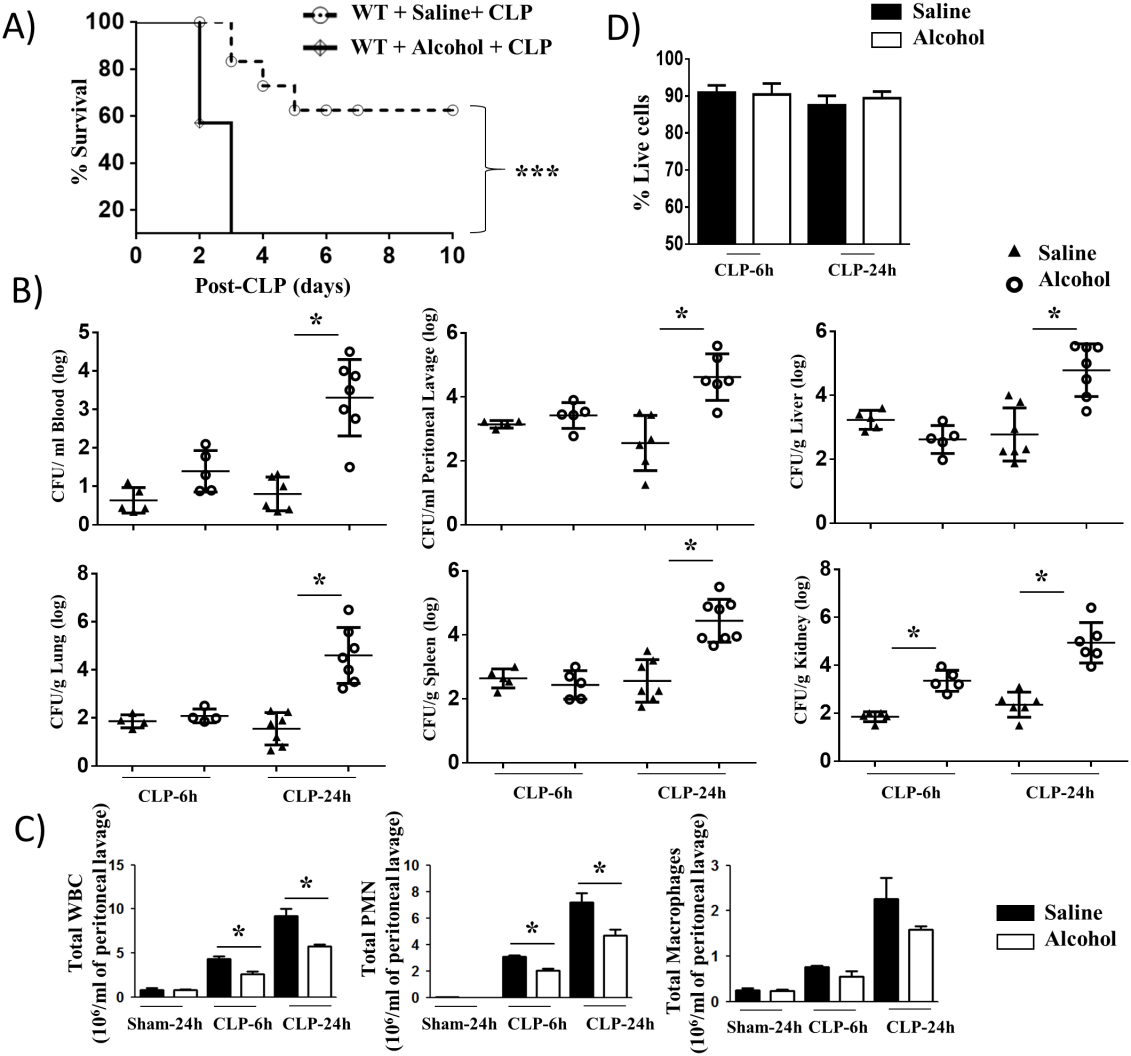
Alcohol intoxication augments susceptibility to CLP and impairs cellular infiltration and bacterial clearance. **A**, Enhanced mortality of acute alcohol intoxicated mice after CLP. Alcohol intoxicated and control (saline-challenged) mice were subjected to CLP or a sham procedure. Survival of mice was assessed every 12 h until 10 days after CLP. Results are expressed as a percentage of 20 animals per group from 2 separate experiments. Significance between groups was examined by Wilcoxon rank test. **B**, Impaired bacterial clearance in alcohol-challenged mice. CFUs were examined in blood, peritoneal lavage or homogenates obtained from livers, lungs, spleens and kidneys of alcohol-challenged and control mice at 6 and 24 h post-CLP. (n = 4-8/group). **C**, Acute alcohol-intoxicated mice showed reduction in total while blood cell count and neutrophil (PMN) and macrophage accumulation in the peritoneum after CLP, as measured by direct cell counts in peritoneal lavage (PL). (n = 4-8/group). **D**. Peritoneal cell (neutrophil) viability was not different between alcohol- and saline-challenged mice. A total of 1000 cells were examined in peritoneal fluid of each group was enumerated using trypan blue exclusion assay (n = 4-6/group). *, *p*<0.05, ***, *p*<0.001.

To explore the potential effect of alcohol on host survival in response to polymicrobial sepsis, C57BL/6 (Wild-type: WT) mice were subjected to CLP-induced sepsis in the presence or absence of acute alcohol administration and the survival of animals was monitored up to 10 days post-CLP. Acute alcohol intoxication caused a reduction in survival compared to saline-challenged mice (Fig 1A). To determine whether a defect in bacterial clearance contributed to the death of mice in the alcohol-administered group, bacterial numbers in peritoneal lavage, blood, spleen, lung, kidney, and liver were enumerated at 6 and 24 h post-CLP. As compared to mice with saline administration, mice administered alcohol had higher bacterial burdens in peritoneal lavage, blood, spleen, lung, kidney, and liver at 24 h post-CLP (Fig 1B), indicating that alcohol facilitates bacterial growth and dissemination in mice with CLP. To further examine the possibility that the increased bacterial burden and higher mortality in mice given alcohol was due to an impaired ability to recruit immune cells such as neutrophils and/or macrophages to the peritoneum, total and differential cell counts in peritoneal lavage were performed. In mice with acute alcohol intoxication, total white blood cell counts and neutrophils, but not macrophages, were decreased both at 6 h and 24 h post-CLP as compared to mice with saline administration (Fig 1C). In mice receiving the sham-operation, no significant cellular influx was observed in the peritoneal lavage of both alcohol-treated and saline-challenged groups (Fig 1C). On an average, >81% of peritoneal cells in peritoneal lavage were neutrophils in saline-challenged and alcohol-treated groups at 24 h post-CLP (Fig 1C). When we determined the viability of peritoneal cells in both alcohol-treated and saline-administered mice, there was no significant difference in viability observed between peritoneal cells (neutrophils) obtained from the saline-challenged and alcohol-treated groups (Fig 1D).

Since CXCL1 is essential for neutrophil recruitment to tissues [17, 18], we determined the CXCL1 levels in serum and peritoneal fluid. Our findings indicate that CXCL1/KC level was reduced in peritoneal fluid, but not in serum, of the alcohol-treated group at 6 h but not at 24 h following CLP (Supp. 1A-B).

### Peritoneal cells (neutrophils) from alcohol-challenged CLP mice display reduced NETosis

**Fig 2:**
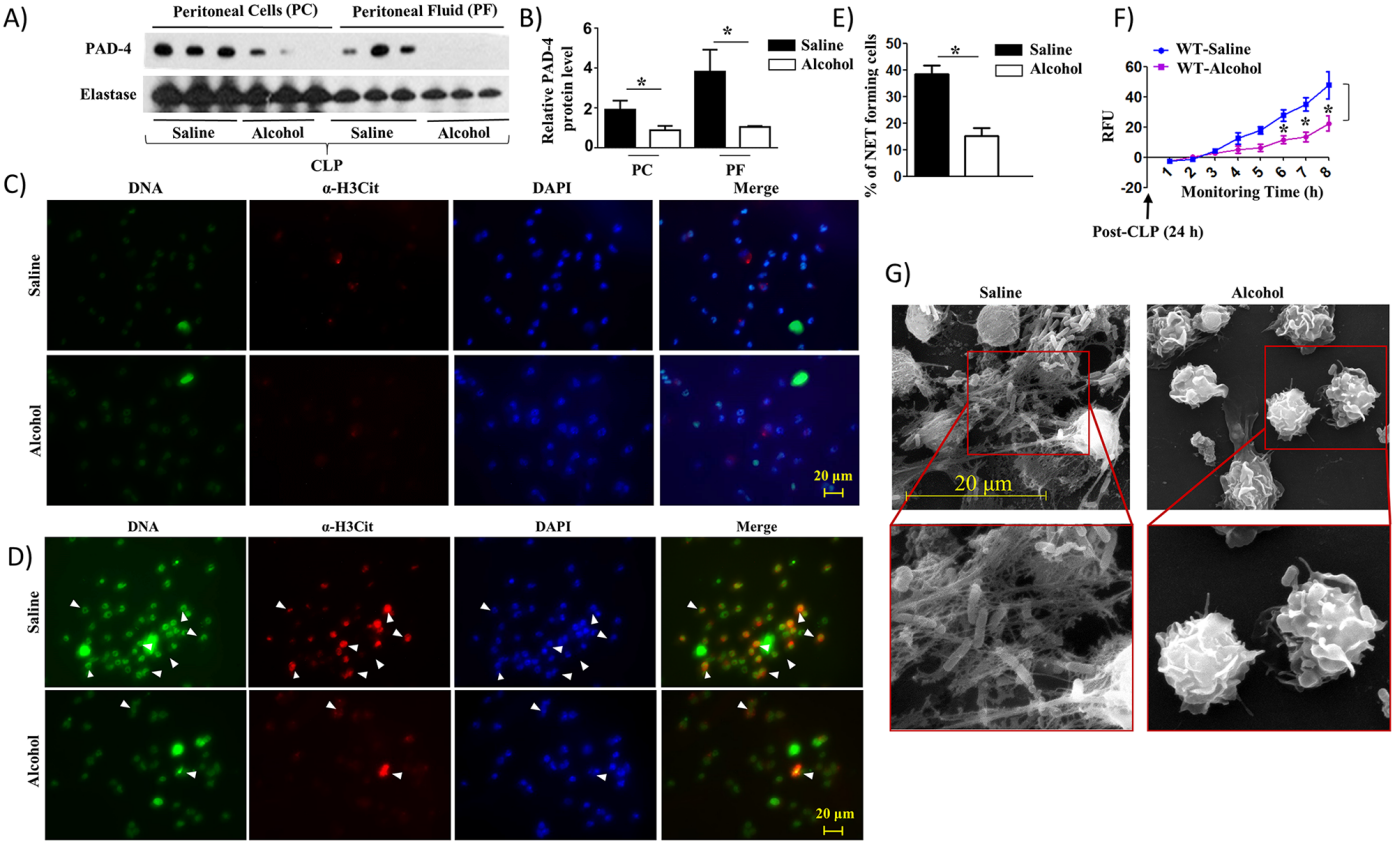
Alcohol impairs NETosis in response to CLP. **A**, Western blot of PAD-4 and neutrophil elastase expression in peritoneal cells (neutrophils) and peritoneal fluid from alcohol-administered and control (saline-challenged) mice at 24 h post-CLP. n = 3 mice/group from two independent experiments. **B**. Relative band densities normalized against neutrophil elastase are representative of the blots. **C-D**, Peritoneal cells (neutrophils) harvested from alcohol-challenged and control (saline-challenged) mice at 24 h post-CLP were washed with saline (0 h), added SYTOX Green and allowed to monitor NETosis for 8 h and then were fixed. Neutrophils were stained with DAPI to stain DNA and citrullinated H3 Ab (α-H3Cit) to visualize citrullinated histone. Double-positive cells are indicated by arrowheads as evidence of NETosis. Images presented are representative of two independent experiments (n = 5-7/group). **E**, Twenty random images were selected from three experiments and quantified for the presence of double-positive (SYTOX Green and H3Cit) peritoneal cells (neutrophils) from alcohol-administered and control mice. n = 5-8/group. **F**, Kinetic analysis of NETosis by peritoneal cells (neutrophils) harvested from alcohol-administered and control mice, washed with saline, and NETosis was measured up to 8 h. Relative fluorescence intensity (RFU) was determined to evaluate NETosis each hour up until 8 h of *in vitro* culture. n = 6-9/group. **G**, Evaluation of NETosis by SEM. Peritoneal cells (neutrophils) harvested from alcohol-administered and control mice after CLP were analyzed by SEM. Presence of long thread-like structures is evidence of NETosis. Images presented are representative of two independent experiments (n = 5-8/group). *, *p*<0.05. NET forming peritoneal cells (neutrophils) are indicated by the arrows on merged images and original magnification 20x. SEM original magnification 8000x.

Although NETosis by neutrophils during infection is a well-established microbial killing mechanism, the effects of alcohol on NETosis remain elusive [19]. To assess this mechanism, we first used western blotting to determine the expression of PAD-4, a protein associated with NETosis [20], in peritoneal cells (neutrophils) and peritoneal fluid obtained from saline-challenged and alcohol-intoxicated septic mice. We found reduced expression of PAD-4 in peritoneal cells (neutrophils) and peritoneal fluid obtained from alcohol-challenged septic mice (Fig 2A) and we quantified the PAD-4 expression using neutrophil elastase as a loading control (Fig 2B). We further assessed NETosis in peritoneal cells (neutrophils) obtained from alcohol-treated and saline-challenged CLP mice using immunofluorescence as described in a previous publication [21]. No NETosis was observed in peritoneal cells (neutrophils) obtained from peritoneal lavage at 24 h post-CLP (Fig 2C) which could be due to isolation procedure of peritoneal cells (neutrophils) from peritoneal lavage because NETs are delicate and filigree structures. However, the fluorescence microscopy results are consistent with the marked reduction in NET forming cells observed at 8 h in peritoneal cells (neutrophils) from alcohol-administered septic mice compared to saline-administered septic mice (Fig 2D and 2E). As shown in Fig 2F, time-dependent NETosis up to 8 h in peritoneal cells (neutrophils) isolated from alcohol-administered septic mice compared to control mice exhibit marked reduction in NETosis in alcohol-treated mice at 6, 7, and 8 h time-points. Utilizing scanning electron microscopy (SEM), long string-like extracellular threads, morphology typical of NETs as defined by Brinkmann *et al*. [22], entangling bacteria were evident in peritoneal cells (neutrophils) from saline-administered septic mice, but not in peritoneal cells (neutrophils) from alcohol-administered septic mice (Fig 2G). We also examined whether alcohol has any effect on the maturation of neutrophils. In this regard, we used transmission electron microscopy (TEM) to detect the presence of primary, secondary and tertiary granules in peritoneal cells (neutrophils) obtained from alcohol-treated and saline-challenged mice. We found no difference in the presence of these granules in peritoneal cells (neutrophils) (dark colored for primary, big and light colored for secondary, and small and light colored for tertiary granules) between saline-challenged and alcohol-treated groups (Supp 2A). Furthermore, the alcohol-exposed peritoneal cells (neutrophils) produce lower levels of antimicrobial peptide, such as cathelicidin at 24 h but not at 6 h, post-CLP (Supp 2B).

### Alcohol-challenged mouse and human neutrophils show attenuated NETosis and NET-mediated killing

**Fig 3:**
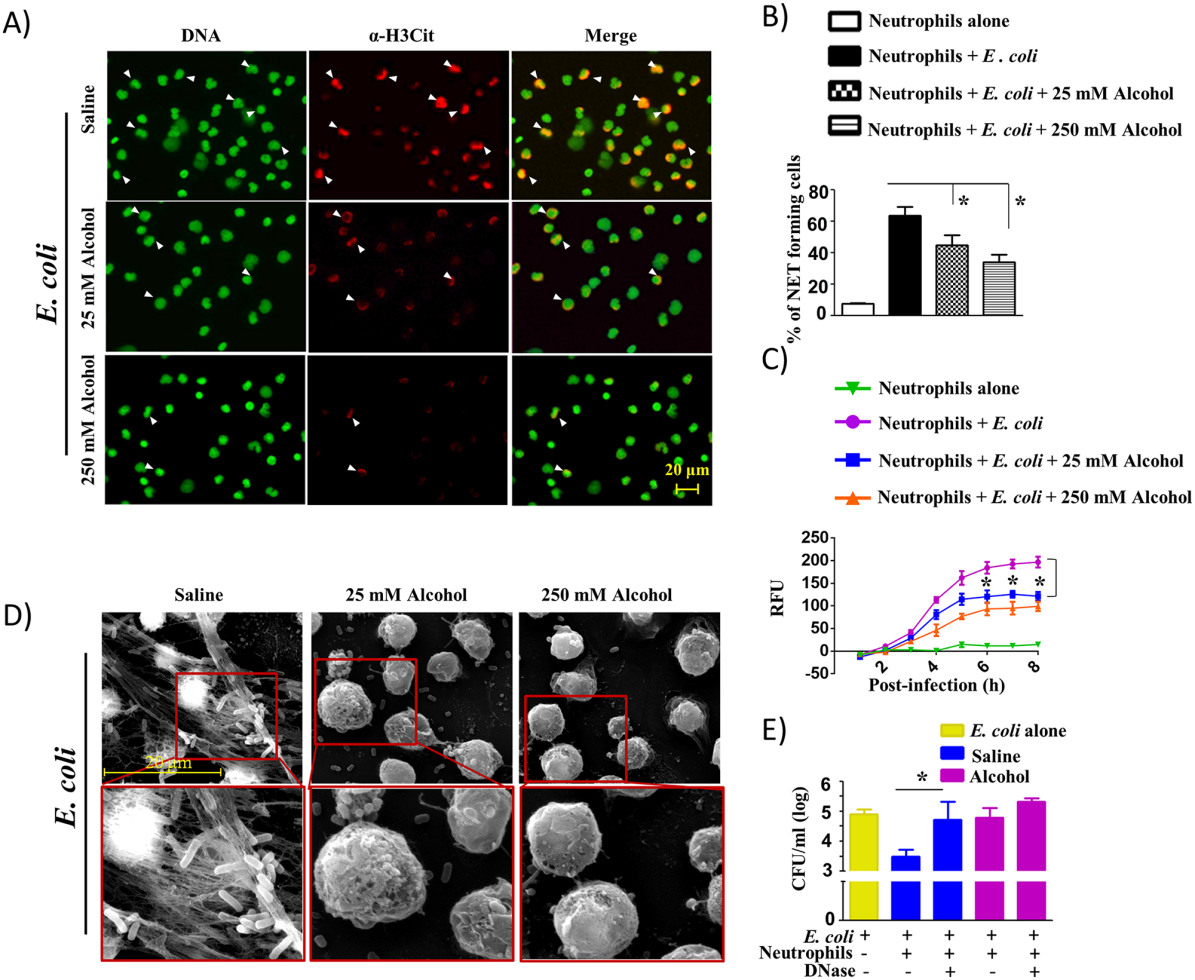
Alcohol reduces NETosis and NET-mediated bacterial killing in mouse bone marrow-derived neutrophils triggered by *E*. *coli* infection. **A**, Mouse neutrophils harvested from WT mice were pretreated with either 25 or 250 mM alcohol before infection with *E*. coli (MOI 1), added SYTOX Green, allowed to undergo NETosis, and then fixed after 8 h. Neutrophils were stained with citrullinated H3 Ab to visualize citrullinated histones in fixed cells. DNA and Citrullinated histones are indicated by arrowheads and double positive cells are considered of NETosis. Images presented are representative of three independent experiments (each in duplicate). **B**, Twenty random images were selected from three experiments and quantified for the presence of NET-positive neutrophils. **C**, Kinetic analysis of NETosis by *E*. *coli*-infected mouse neutrophils treated with alcohol. Mouse neutrophils were pretreated with either 25 mM or 250 mM alcohol and infected with *E*. *coli* and stained with SYTOX Green DNA stain to visualize every hour over a period of 8 h. Relative fluorescent intensity (RFU) was determined to evaluate NETosis. Significant differences between infected and alcohol-treated (25 mM alcohol) are indicated by asterisks. **D**, Evaluation of NETosis by SEM. Mouse neutrophils were pretreated with either 25 or 250 mM alcohol before infection with *E*. *coli* and incubation for 8 h to allow for NETosis. **E**, Alcohol-treated mouse bone marrow neutrophils exhibited diminished NET-mediated killing activity. Bacterial killing capacity of *E*. *coli*-infected, alcohol-treated and untreated bone marrow neutrophils was determined by assessing extracellular (CFUs) at 8 h post-infection with *E*. *coli* (MOI 1) in the presence or absence of DNase. For experiments A-E, four to five mice/group were used and cells used in triplicate in 3 separate experiments, *, *p*<0.05. NET forming neutrophils are indicated by the arrows on merged images and original magnification 20x. SEM original magnification 8000x.

**Fig 4:**
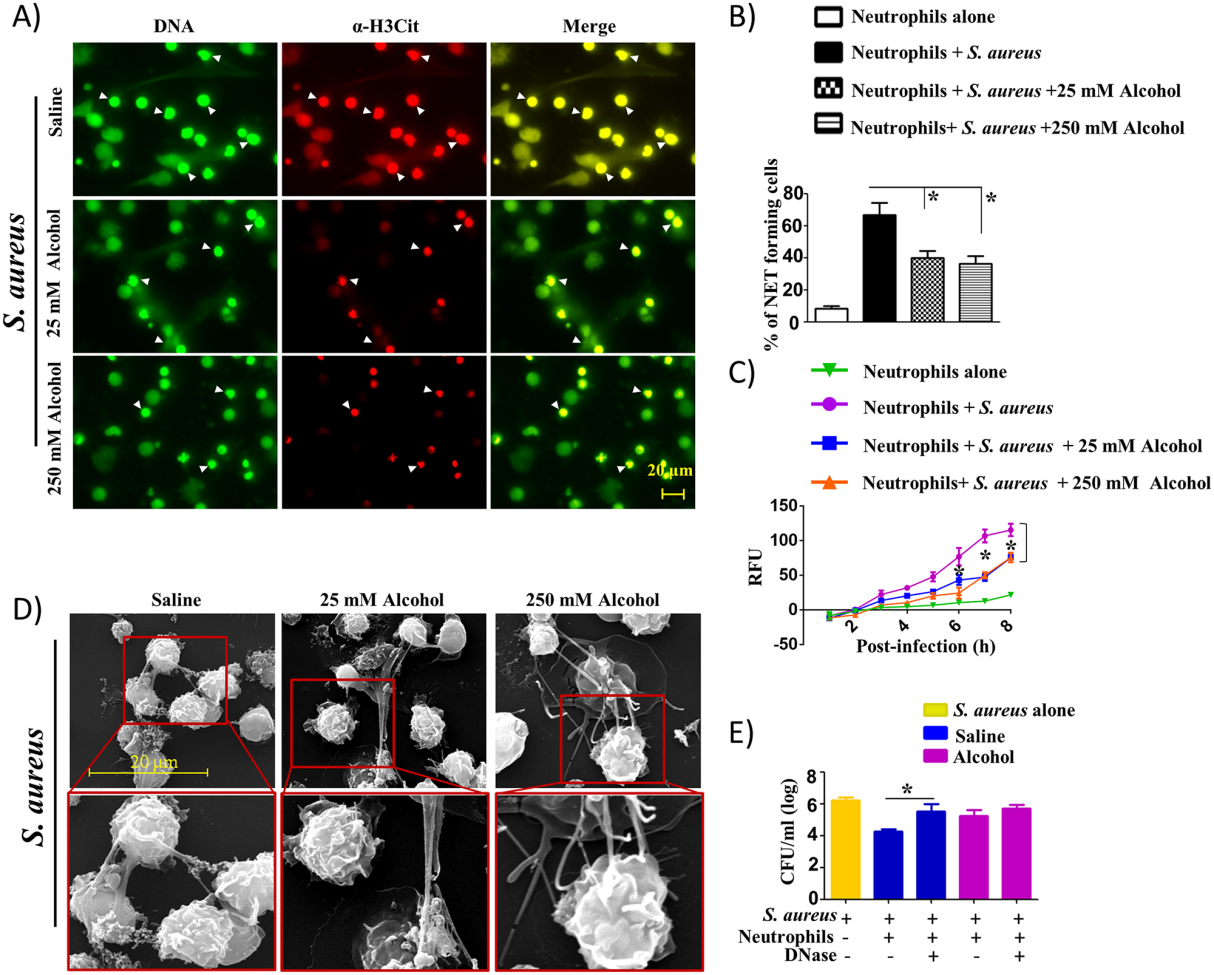
Alcohol decreases NETosis and NET-mediated bacterial killing in mouse bone marrow-derived neutrophils in response to *S*. *aureus* infection. **A**, Mouse bone marrow-derived neutrophils treated with 25 or 250 mM alcohol exhibit diminished NETosis in response to Gram-positive bacterial (*S*. *aureus*) infection. Mouse neutrophils harvested from C57BL/6 mice were pretreated with either 25 or 250 mM alcohol before infection with *S*. *aureus* (MOI 1), added SYTOX Green, monitored NETosis, and then fixed after 8 h. Neutrophils were stained with citrullinated H3 Ab to visualize citrullinated histones after the cells were fixed with 4% paraformaldehyde. Double-positive cells are indicated by arrowheads as evidence of NETosis. **B**, Twenty images in a random manner were selected from three experiments and quantified for the presence of NET-positive neutrophils. **C**, Kinetics of NETosis by *S*. *aureus*-infected mouse neutrophils treated with alcohol. Mouse neutrophils were pretreated with either 25 mM or 250 mM alcohol and infected with *S*. *aureus* and stained with SYTOX Green DNA stain to visualize NET formation every hour over a 8 h period. Relative fluorescent intensity (RFU) was determined to determine NETosis. Significant differences between infected and alcohol-treated (25 mM alcohol) are indicated by asterisks. **D**, Assess NETosis by SEM. Mouse neutrophils were pretreated with either 25 or 250 mM alcohol before infection with *S*. *aureus* and incubation for 8 h to allow for NETosis. **E**, Alcohol-treated mouse bone marrow neutrophils show diminished NET-mediated killing activity. Bacterial killing capacity of *S*. *aureus*-infected, alcohol-treated and untreated bone marrow-derived neutrophils was determined by assessing extracellular (CFUs) at 8 h post-infection with *S*. *aureus* (MOI 1) in the DNase treated and untreated groups. Experiments A-E were performed in triplicate and 4–5 mice/group were used. *, *p*<0.05. NET forming neutrophils are indicated by the arrows on merged images and original magnification 20x. SEM original magnification 8000x.

Next, we determined the effect of alcohol on NETosis in mouse and human neutrophils (*in vitro*) following bacterial infection. Regarding the concentration of alcohol for the *in vitro* tissue culture, concentrations between 10 and 500 mM have been used. The 25 mM *in vitro* concentration is equivalent to 0.1 g/dl blood alcohol reached in healthy (nonalcoholic) people following 4–5 drink equivalents termed “moderately drunk” situation [23] While the 25 mM concentration corresponds to a blood alcohol concentration of ~0.1%, which is encountered commonly in the clinical setting and is just above the legal intoxication threshold, a concentration of 250 mM corresponds to a blood alcohol concentration of 1%, which is 12 times the legal threshold [24]. For these experiments, we analyzed NETosis against *E*. *coli* (Gram-negative) and *S*. *aureus* (Gram-positive) in bone marrow-derived neutrophils obtained from wild-type (C57BL/6) mice at a multiplicity of infection (MOI) of 1 in the presence or absence of alcohol (25 mM and 250 mM). The results showed that the percentage of neutrophils positive for extracellular DNA (SYTOX Green) and citrullinated histone H3 decreased with alcohol treatment of bone marrow-derived neutrophils following *E*. *coli* (Fig 3A and 3B) and *S*. *aureus* infection (Fig 4A and 4B). The reduction of NETs by alcohol was observed to be consistent over time course of 8 h incubation as relative fluorescence units (RFU) of the alcohol-treated cells positive for extracellular DNA (SYTOX Green) were decreased continuously over the 8 h time window (Figs 3C and 4C). In addition, SEM images corroborate the reduced formation of NETs by alcohol-treated, bone marrow-derived neutrophils at 8 h post-infection (Figs 3D and 4D). Since SYTOX Green stains extracellular DNA, we also used DAPI to stain intracellular DNA to demonstrate that SYTOX Green colocalizes with DAPI (Supp 3) to confirm specific staining of cells. Furthermore, we have performed additional experiments in bone marrow-derived mouse neutrophils to demonstrate that alcohol inhibits NETosis induced by PMA (a non-infectious stimulus) and found that PMA-induced NETosis was also attenuated by alcohol exposure at 8 h (Supp 4A-B).

Consistent with the NETosis observed in bone marrow-derived neutrophils (BMDNs) from saline challenged and alcohol-treated groups (Figs 3A–3D and 4A–4D), we finally determined NET-mediated bacterial killing in BMDNs following alcohol challenge and infection with either *E*. *coli* or *S*. *aureus*. We specifically used DNase in these experiments because DNase degrades NETs. In BMDNs after alcohol challenge (*in vitro*), we found no significant decrease in NET-mediated bacterial killing of Gram-negative (*E*. *coli*) and Gram-positive (*S*. *aureus*) bacteria at 8 h post-infection between the presence and absence of DNase (Figs 3E and 4E). However, in BMDNs after saline challenge, we found a significant decrease in NET-mediated bacterial killing of Gram-negative (*E*. *coli*) and Gram-positive (*S*. *aureus*) bacteria at 8 h post-infection following DNase treatment as compared to the absence of DNase (Figs 3E and 4E).

To examine whether the impairment of bacteria-induced NETosis and NET-mediated bacterial killing by alcohol can be observed in human neutrophils, we used human neutrophils derived from peripheral blood of healthy volunteers. Similar to the results seen in peritoneal cells (neutrophils) from CLP-mice and in BMDNs treated with alcohol, the percentage of neutrophils positive for both extracellular DNA (SYTOX Green) and citrullinated histone H3 was lower in 25 mM and 250 mM alcohol-treated human neutrophils following *E*. *coli* (Supp 5A-B) or *S*. *aureus* infection (Supp 6A-B). Moreover, kinetic analysis of NETs conducted over a 8 h time period showed a reduced level of NETosis by alcohol-treated human neutrophils at 7 h and 8 h time points as compared to saline-treated human neutrophils infected with either *E*. *coli* (Supp 5C) or *S*. *aureus* (Supp 6C). In a similar manner, SEM images demonstrate markedly reduced or absent NETosis by alcohol-treated human neutrophils infected with *E*. *coli* (Supp 5D) or *S*. *aureus* (Supp 6D) at both concentrations of alcohol (25 mM and 250 mM). DNase treatment (to degrade NETs) shows the important role of NET-mediated killing of *E*. *coli* (Supp 5E) and *S*. *aureus* (Supp 6E) by human neutrophils at 8 h post-infection in saline-challenged group.

### Alcohol reduces ROS production in mouse peritoneal cells (neutrophils) and bone marrow-derived neutrophils

**Fig 5:**
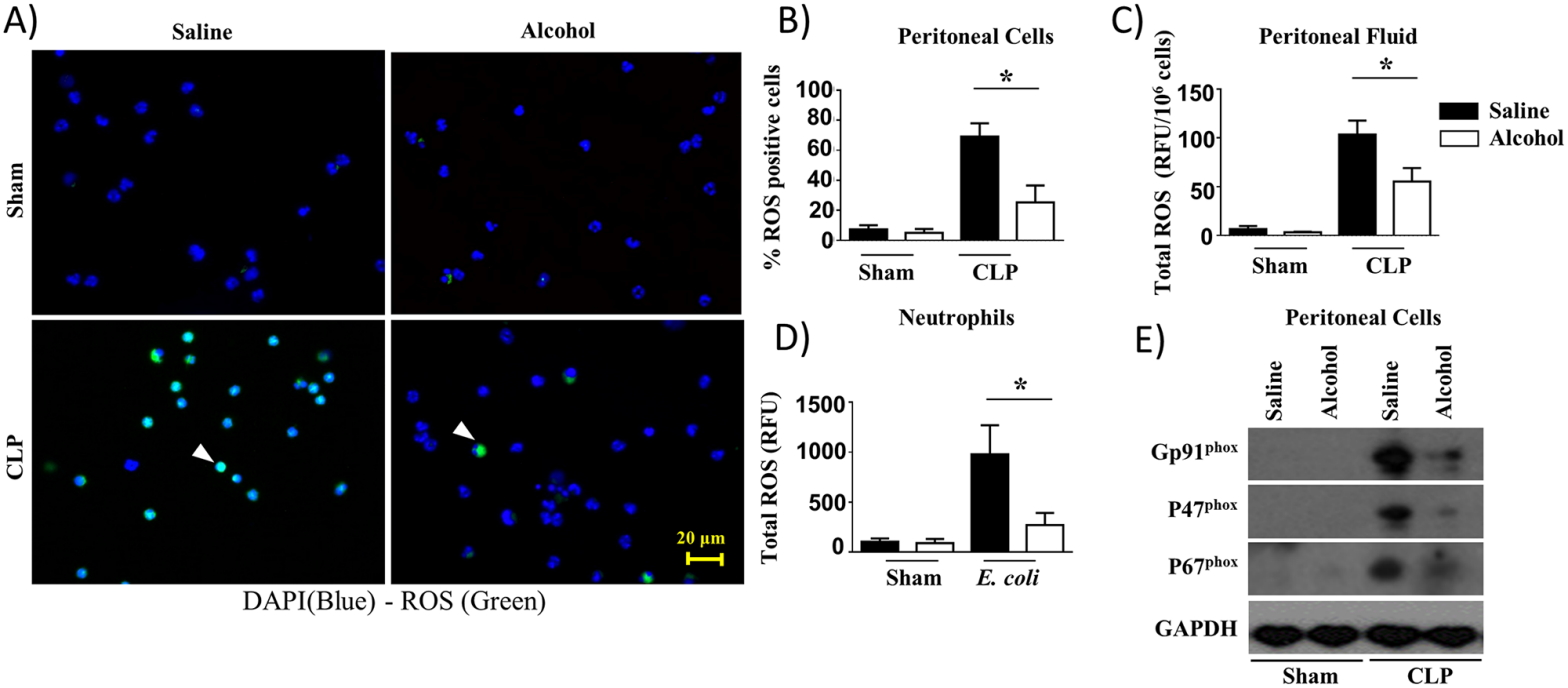
Attenuation of ROS production in alcohol-challenged mice and in alcohol-treated bone marrow-derived neutrophils in response to bacterial infection. **A**, ROS^+^ neutrophils were identified by fluorescence microscopy after intracellular staining for ROS in peritoneal cells (neutrophils) derived from alcohol-administered and control mice at 24 h post-CLP. Results are representative of a microscopic view of three independent experiments (ROS+ cells are green, ROS^−^ cells are blue). Arrows show ROS producing cells. **B**, Twenty random images were selected from three experiments and quantified for the presence of ROS-positive (green) neutrophils. (*, *p*<0.05). **C**, The production of total ROS in peritoneal fluid of alcohol-administered and control mice at 24 h post-CLP were evaluated as relative fluorescence units (RFUs) and normalized to total peritoneal cells. **D**. Total ROS production by bone marrow-derived neutrophils stimulated with *E*. *coli* were quantified as relative fluorescence units (RFU). (n = 5-8/group; *, *p*<0.05). **E**. Western blotting of GP91^phox^, P47^phox^, P67^phox^ and GAPDH expression in peritoneal cells (neutrophils) from alcohol-administered and saline-challenged mice at 24 h post-CLP. This is a representative blot of 3 independent experiments with identical results. Original magnification 20x.

We next determined whether defective NETosis observed in peritoneal cells (neutrophils) from CLP mice and in neutrophils infected with bacteria following alcohol challenge is due to a reduction in ROS production. To assess this mechanism, ethanol was administered to mice prior to CLP and peritoneal cells (neutrophils) and peritoneal fluid were analyzed for ROS production at 24 h after CLP. Neutrophils purified from the bone marrow were also used for ROS determination at 8 h post-infection with *E*. *coli*. The results of these experiments illustrate that alcohol administration in mice impairs the production of ROS in peritoneal cells (neutrophils) following CLP (Fig 5A and 5B). The reduced percentage of ROS^+^ cells among total peritoneal cells (neutrophils) is consistent with the finding of diminished ROS levels in peritoneal fluid at 24 h post-CLP (Fig 5C). These *ex vivo* results were confirmed by total ROS production by BMDNs following *E*. *coli* infection at 8 h (Fig 5D). To explore if NADPH oxidase is essential for ROS generation in the peritoneum following CLP, we examined the expression of Gp91^phox^, P47^phox^ and P67^phox^ in peritoneal cells (neutrophils) at 24 h. Our findings indicate substantial reduction in the expression of the components of NADPH oxidase at 24 h post-CLP (Fig 5E).

### Alcohol attenuates extracellular bacterial killing of mouse and human neutrophils

**Fig 6:**
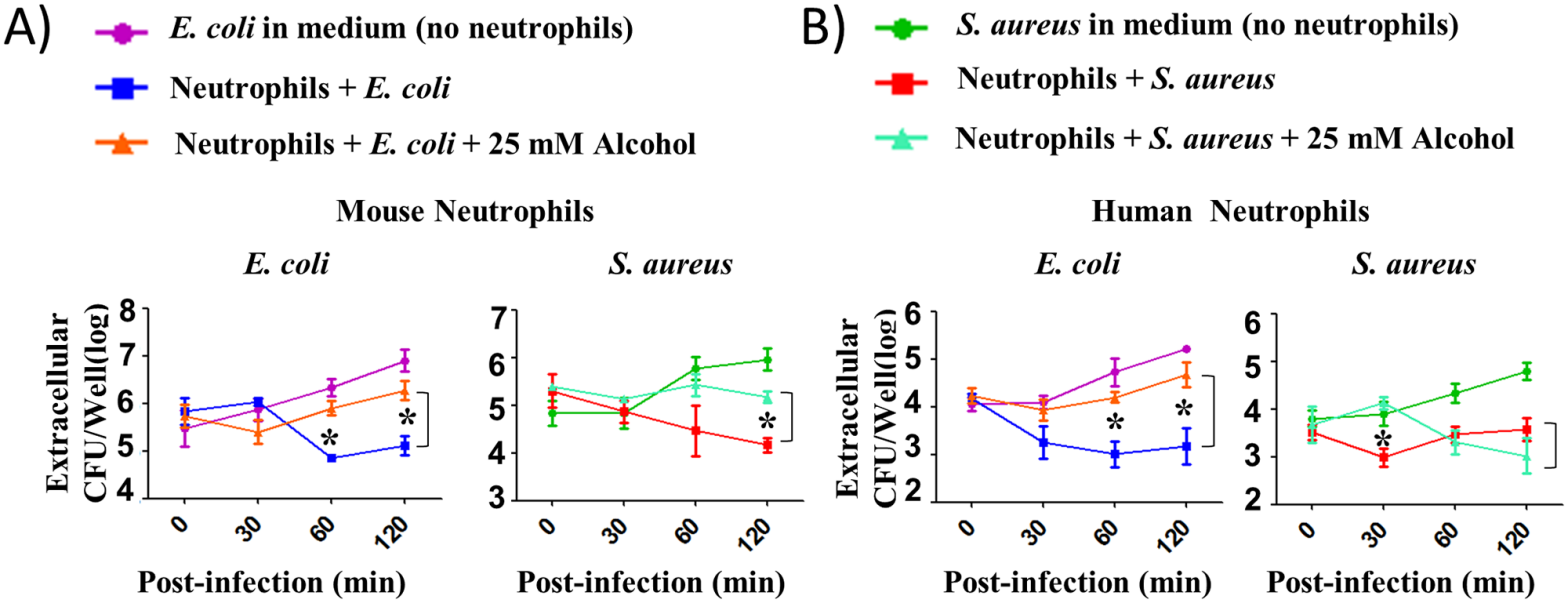
Alcohol impairs extracellular bacterial killing of mouse and human neutrophils. **A-B**, Bacterial killing capacity of mouse bone marrow-derived neutrophils (A) and human peripheral blood-derived neutrophils (B) pretreated without or alcohol (25 mM) and infected with an MOI of 1 was determined by assessing extracellular CFUs at 30, 60, and 120 min after infection with *E*. *coli* or *S*. *aureus*. Experiments were performed in triplicate wells and from three independent experiments and 3–5 mice/group was used. For human neutrophils, 4–5 donors were used per group (*p <0.05 between infected groups in the presence and absence of alcohol).

To examine whether alcohol impairs extracellular killing mechanisms of mouse and human neutrophils, we performed bacterial killing assays against *E*. *coli* and *S*. *aureus*. For these assays, BMDNs and human peripheral blood-derived neutrophils were first infected with either *E*. *coli* or *S*. *aureus* at an MOI of 1 for extracellular killing with or without treatment with 25 mM alcohol. The extracellular killing of neutrophils was assessed by enumerating bacteria in supernatants. The results show that alcohol inhibits the extracellular killing ability of both mouse (Fig 6A) and human neutrophils (Fig 6B), as we observed significantly higher numbers of viable extracellular bacteria in the wells of alcohol-treated mouse and human neutrophils at 30, 60 or 120 min post-infection (Fig 6A and 6B).

### rCXCL1 rescues host defense in acute alcohol intoxicated septic mice

**Fig 7:**
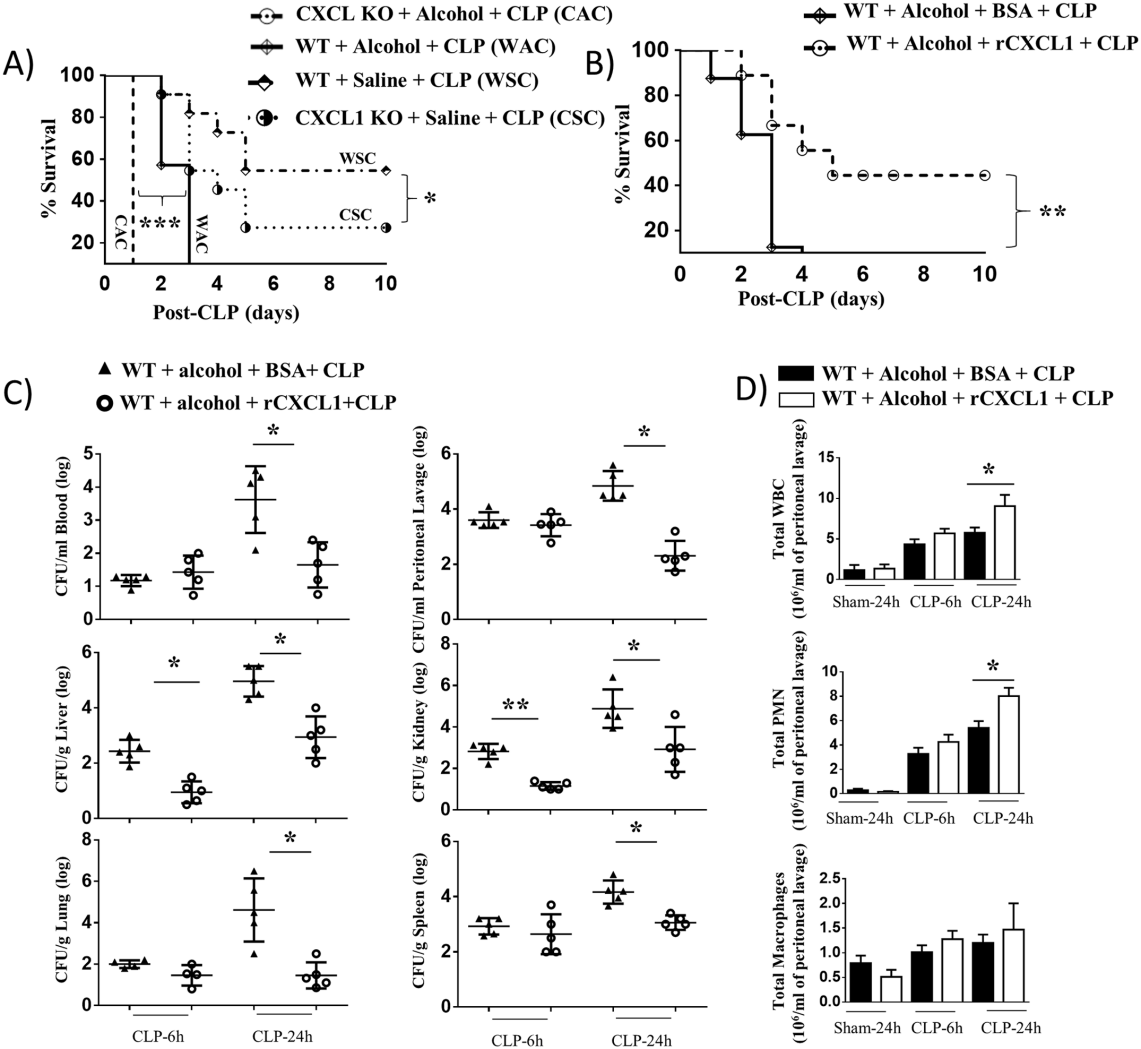
rCXCL1 reduces susceptibility to alcohol-challenged CLP mice and augments bacterial clearance and neutrophil recruitment. **A**, Enhanced mortality of alcohol intoxicated WT and CXCL1 knockout (KO) mice following CLP. Alcohol intoxicated and control (saline-challenged) mice were subjected to CLP. Survival of mice was assessed every 12 h until 10 days after CLP. Results are expressed as a percentage of 20 animals per group. Significance between groups was examined by Wilcoxon rank test. WSC versus CSC (*, *p*<0.05), WAC versus CAC (***, *p*<0.001), WAC versus WSC (***, *p*<0.001), and CAC versus CSC (***, *p*<0.001). **B**, Reduced mortality of alcohol treated WT mice by rCXCL1 following CLP. Alcohol intoxicated WT mice were subjected to CLP followed by rCXCL1 or BSA treatment. Survival of mice was assessed every 12 h until 10 days after CLP. Results are expressed as a percentage of 20 animals per group. Significance between groups was examined by Wilcoxon rank test. **, *p*<0.01. **C**, Impaired bacterial clearance in alcohol-challenged mice. CFUs were examined in peritoneal lavage and blood or homogenates obtained from kidneys, livers, lungs, and spleens of alcohol-challenged and control mice at 6 and 24 h post-CLP. (n = 6/group). **D**, Acute alcohol-intoxicated mice showed reduced total while blood cell count and neutrophil (PMN) and macrophage influx in the peritoneum after CLP, as measured by direct cell counts in peritoneal lavage (PL). (n = 4-8/group). *, *p*<0.05.

To explore the role of CXCL1 in host defense in response to acute alcohol challenge followed by CLP, we used WT mice and CXCL1 KO mice and the survival was monitored up to 10 days post-CLP in the presence of saline or alcohol. As indicated in Fig 7A, acute alcohol intoxication caused a substantial reduction in survival in both WT and CXCL1 KO mice following CLP. In addition, there was a substantial reduction in survival in alcohol-treated WT mice (WAC) as compared with saline-treated WT mice (WSC) following CLP (*p*<0.001). In a similar manner, alcohol-treated CXCL1 KO (KO) mice (CAC) display significant reduction in survival than saline-treated CXCL1 KO mice (CSC) following CLP (*p*<0.001). Moreover, significant difference in survival was observed between alcohol-treated WT mice and alcohol-administered CXCL1 KO mice. To further determine the effect of rCXCL1 on survival in response to alcohol-challenge followed by CLP, WT mice were subjected to alcohol challenge followed by CLP in the presence of rCXCL1 or BSA and the survival of mice was monitored up to 10 days. As shown in Fig 7B, rCXCL1 administration caused an increase in survival compared to control (BSA-administered) WT mice. To explore whether an improvement in bacterial clearance contributed to the survival of mice in the rCXCL1-administered group, bacterial numbers in peritoneal lavage, blood, spleen, lung, kidney, and liver were enumerated at 6 and 24 h post-CLP. As compared to mice with BSA administration, mice administered rCXCL1 had lower bacterial burden in peritoneal lavage, blood, spleen, kidney, lung, and liver examined (Fig 7C), indicating that rCXCL1 restricts bacterial growth and dissemination in acute alcohol-challenged CLP mice. To further examine that the decreased bacterial burden and lower mortality in mice given rCXCL1 was due to an impaired ability to recruit immune cells, such as neutrophils and/or macrophages to the peritoneum, total and differential cell counts in peritoneal lavage were enumerated. In mice with rCXCL1 administration, total white blood cell counts and neutrophils, but not macrophages, were increased in peritoneal lavage at 24 h post-CLP as compared to mice with BSA administration in alcohol-treated mice (Fig 7D). In mice that underwent the sham-operation, no substantial cellular (neutrophil) influx was observed in the peritoneal lavage of either group (Fig 7D).

### Recombinant CXCL1 (rCXCL1) augments NETosis in peritoneal cells (neutrophils) isolated from alcohol-challenged septic mice

**Fig 8:**
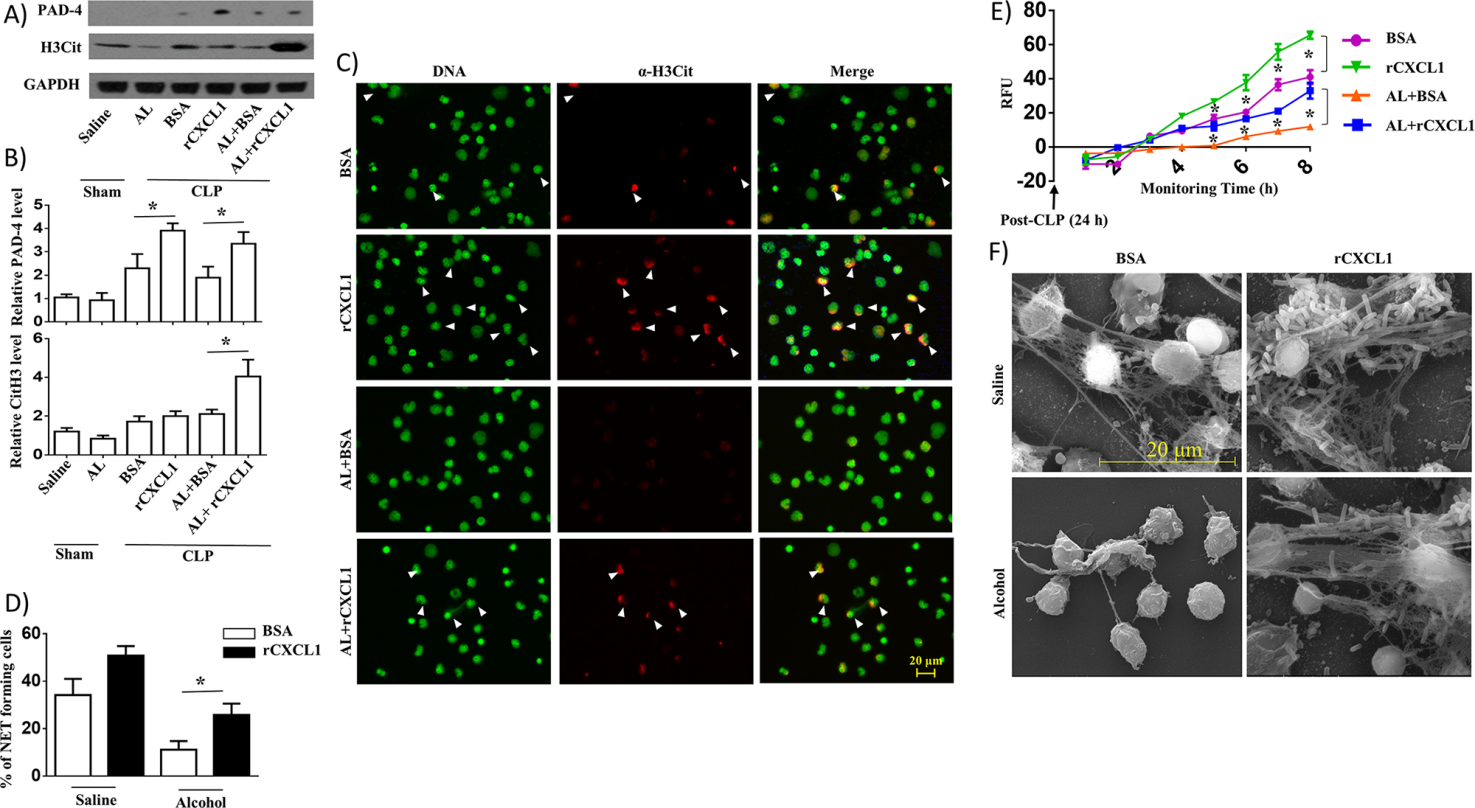
rCXCL1 enhances NETosis in alcohol-challenged septic mice. **A**, Western blotting of PAD-4, H3Cit and GAPDH in peritoneal cells (neutrophils) from alcohol-administered and control mice at 24 h post-CLP after rCXCL1 or BSA administration. This blot is representative of three independent experiments with identical results. **B**. Relative densities normalized against GAPDH are representative of the blots from 3 separate experiments (n = 3-5/mice/group). **C**, Peritoneal cells (neutrophils) harvested from alcohol-challenged and control mice after CLP were allowed to undergo NETosis after adding SYTOX Green for 8 h and then were fixed. Neutrophils were stained with citrullinated H3 Ab (α-H3Cit) to visualize citrullinated histone. Double-positive cells are indicated by arrowheads as evidence of NETosis. Images presented are representative of three independent experiments (n = 5-7/group). **D**, Twenty random images were selected from three experiments and quantified for the presence of NET-positive (double-positive; SYTOX Green- and H3Cit-positive) neutrophils from alcohol-administered and control mice. n = 5-8/group. **E**, Kinetic analysis of NETosis by peritoneal cells (neutrophils) harvested from alcohol-administered and control mice. Relative fluorescence intensity (RFU) was determined to evaluate NETosis each hour up until 8 h of culture. n = 6-9/group. Significance was calculated between rCXCL1 treated and BSA treated peritoneal cells from mice in the presence or absence of alcohol. **F**, Evaluation of NETosis by SEM. Peritoneal cells (neutrophils) harvested from alcohol-administered and control mice after CLP in rCXCL1 or BSA administration were analyzed by SEM. Presence of long thread-like structures (arrowhead) is evidence of NETosis. Scale bars, 20 μm. (n = 5–8 mice/group); NET forming neutrophils are indicated by the arrows and original magnification 20x; *, *p*<0.05. AL, alcohol. Arrow indicates NET forming cells on merged images. SEM original magnification 8000x.

To determine the role of CXCL1 in NETosis, we used western blotting to determine the expression of PAD-4 and citrullinated histone 3 (H3Cit), proteins associated with NETosis in peritoneal cells (neutrophils) obtained from saline-challenged and alcohol-intoxicated mice at 24 h post-CLP. The data display augmented expression of PAD-4 and CitH3 proteins in peritoneal cells (neutrophils) from alcohol-treated CLP mice as well as saline-challenged CLP mice administered with rCXCL1 as compared with BSA-treated mice (Fig 8A and 8B). We also assessed NETosis in peritoneal cells (neutrophils) isolated from alcohol-challenged CLP mice using immunofluorescence microscopy. Fluorescence microscopy results indicated a marked reduction in NETosis observed at 8 h in peritoneal cells (neutrophils) isolated from alcohol-administered CLP mice and NETosis was enhanced by rCXCL1 administration in the alcohol-treated mice (Fig 8C and 8D). We also determined time-dependent NETosis up to 8 h in peritoneal cells (neutrophils) isolated from alcohol-administered CLP mice after rCXCL1 administration. We found a marked increase in NETosis at 5, 6, 7 and 8 h in rCXCL1 administered mice as compared to BSA-challenged mice (Fig 8E). Scanning electron microscopy (SEM) images revealed more long string-like extracellular threads, and entangled bacteria were evident in peritoneal cells (neutrophils) isolated from saline-administered, but not alcohol-treated, CLP mice at 24 h. Administration of rCXCL1augmented NETosis in neutrophils from both saline- and alcohol-administered CLP mice at 24 h post-CLP (Fig 8F).

### rCXCL1 augments extracellular bacterial killing by mouse neutrophils

**Fig 9:**
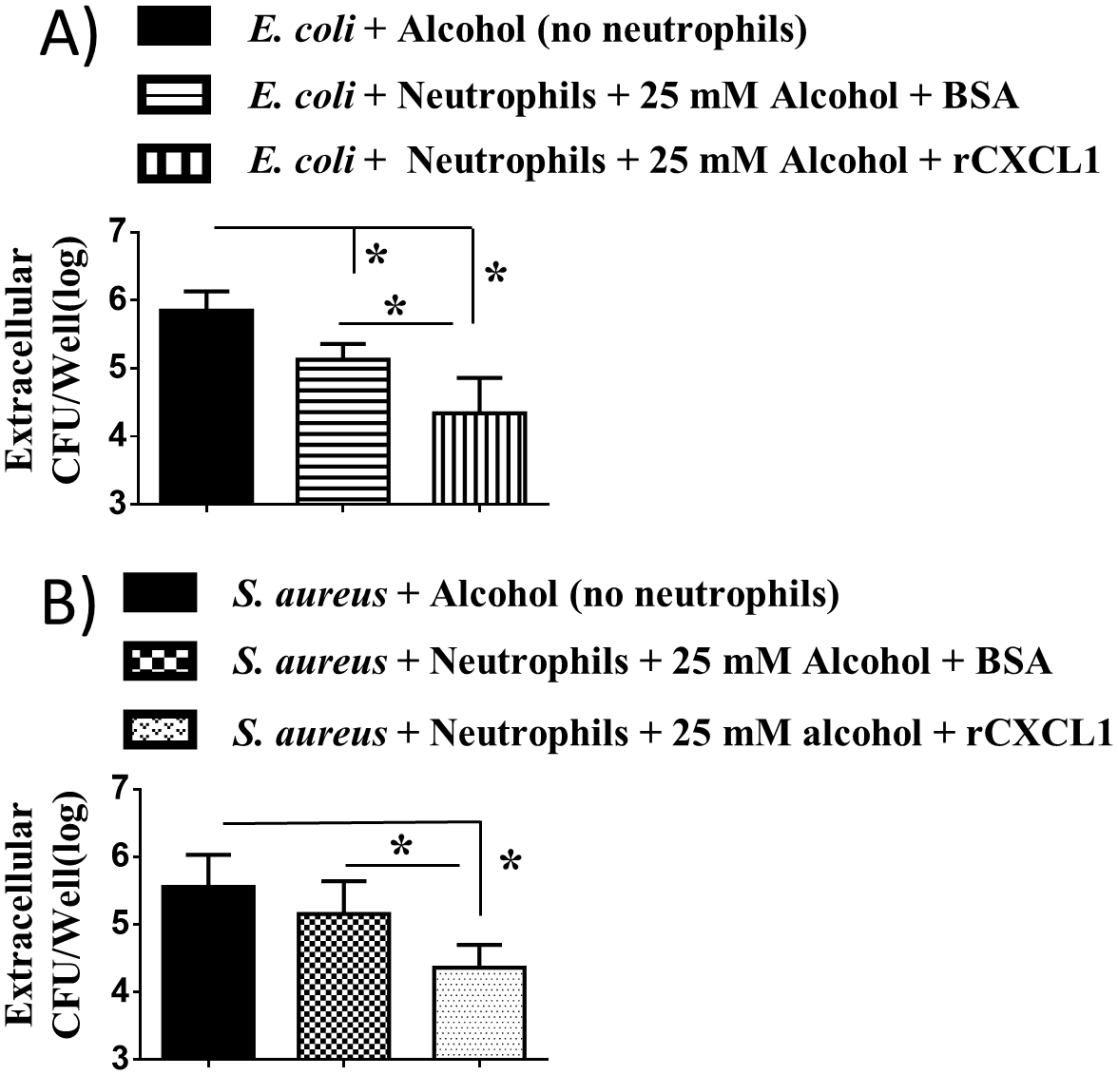
rCXCL1 augments extracellular bacterial killing of neutrophils. **A-B**, Alcohol impairs the extracellular bacterial killing activity of mouse bone marrow-derived neutrophils which can be improved by rCXCL1. Bacterial killing of mouse neutrophils treated with alcohol (25 mM) prior to infection (MOI 1) was determined by assessing extracellular CFUs at 60 min after infection with *E*. *coli* **(A)** or *S*. *aureus* **(B)**. Experiments were performed in triplicate wells and 3–5 mice/group were used (*, *p* <0.05).

In an attempt to examine whether rCXCL1 augments extracellular bacterial killing ability of mouse bone marrow-derived neutrophils, we performed bacterial killing assays against *E*. *coli* and *S*. *aureus*. For these assays, bone marrow-derived neutrophils were treated with 5 nM rCXCL1 or BSA for one hour and infected with either *E*. *coli* (Fig 9A) or *S*. *aureus* (Fig 9B) at an MOI of 1 in the presence of 25 mM alcohol. The killing ability of neutrophils was determined by counting extracellular bacteria. The results show that rCXCL1 enhances bacterial killing of mouse neutrophils in *E*. *coli* and *S*. *aureus*.

## Discussion

The generation of the host immune response to polymicrobial sepsis is a multistep process involving exaggerated inflammation and immune suppression on both the cellular and molecular levels. While NETosis is clearly beneficial as a critical host defense mechanism, uncontrolled NETosis may induce excessive organ damage. It is well known that alcohol consumption alters the immune response and predisposes the host to a variety of Gram-negative and Gram-positive bacterial infections [22, 25, 26]. Although the underlying mechanisms by which alcohol causes immune dysfunction is still not completely understood, acute ethanol intoxication is known to suppress neutrophil recruitment to the lung, neutrophil phagocytosis, and oxidative burst [27]. In addition, it is unclear whether recombinant CXCL1, a neutrophil chemoattractant, can rescue impaired host defense mechanisms, including NETosis in alcohol challenged septic mice. Our studies demonstrate for the first time that NETosis is attenuated and bacterial elimination reduced in acute alcohol-challenged CLP-mice. Furthermore, we used mouse bone marrow-derived and human peripheral blood-derived neutrophils to illustrate that NETosis and NET-mediated killing are attenuated in alcohol treated cells. Moreover, recombinant CXCL1 rescues impaired host defense in acute alcohol challenged septic mice.

When exposed to alcohol, mice exhibited a reduction in total leukocyte and neutrophil numbers in the peritoneum after CLP, and a reduction in bacterial clearance from the peritoneum and extraperitoneal organs, such as lungs, kidney, and spleen. Furthermore, the increased bacterial burden and dissemination is consistent with the reduced survival of mice after acute alcohol intoxication. These findings are in agreement with a previous study, which showed an impairment of neutrophil recruitment to lung and enhanced mortality among alcohol-recipient mice in response to pulmonary *K*. *pneumoniae* infection [28]. In addition to neutrophil influx, neutrophil function is a critical step for suppression of bacterial growth and dissemination in the peritoneum during CLP-induced polymicrobial sepsis [8, 11].

NETosis is a unique host defense mechanism employed by neutrophils [13]. The critical feature specific to NETs is the extrusion of neutrophil nuclear DNA fibers in the extracellular space [13]. NETs are formed by neutrophils upon contact with microbes, such as bacteria, fungi, viruses and protozoa, in order to trap and kill extracellular pathogens [13]. In the present study, we found a reduction in NETosis among acutely alcohol-intoxicated mice following polymicrobial sepsis when compared to control mice, as determined by reduced NET-positive peritoneal cells (neutrophils) by immunofluorescence, the expression levels of PAD-4 protein, and the formation of NET-like structures (by SEM). In addition, our *in vitro* results utilizing mouse bone marrow-derived neutrophils and human neutrophils showed reduced NETosis by alcohol-treated neutrophils exposed to bacteria as compared to neutrophils treated with saline. Furthermore, we demonstrated that diminished NETosis using DNase caused attenuated NET-mediated killing of bacteria. Although this is the first investigation to demonstrate the role of alcohol in NETosis, future studies are required to demonstrate the minimum concentration of alcohol which is required to attenuate or abolish NETosis. Alcohol-induced impairment in NET forming is not specific to infectious stimuli, such as bacteria because of the inhibition in NETosis to a non-infectious stimulus, such as phorbol 12-myristate 13-acetate (PMA).

Reactive oxygen species, which are generated by the activity of phagocytic NADPH oxidase, are a key component of innate immunity and antibacterial defense, but can also be a harmful mediator of acute inflammation when produced in excessive amounts [29]. Although studies have suggested the host deleterious role of ROS in an experimental model of sepsis, it was unknown whether alcohol can alter ROS production causing an effect on bacterial multiplication in mice with polymicrobial sepsis. In this context, our results of decreased NADPH expression, impaired ROS production and enhanced bacterial burden after ethanol exposure suggest that NADPH-dependent ROS production by ethanol is a critical mechanism underlying enhanced bacterial burden followed by polymicrobial sepsis. Consistent with our results, recent studies showed that ethanol significantly decreases ROS production by *Acinetobacter baumannii*-infected neutrophils [30]. Along this line, studies have demonstrated attenuated ROS generation along with increased susceptibility of bacterial infection in mice that have targeted disruptions of NADPH oxidase components (gp91^*phox*^, p47^*phox*^), highlighting the important role of ROS in restricting bacterial multiplication *in vivo* [31, 32]. Our studies confirm the findings from previous studies that alcohol impairs bacterial clearance by reducing the production of ROS through decreasing the NADPH activity [20].

The results linking diminished neutrophil ROS production with attenuated NETosis and subsequent reduction in bacterial killing are consistent with prior reports [22, 33, 34], although the effect of alcohol intoxication on these processes has not been investigated. Our results of the dampening role of alcohol on the ability of neutrophils to kill Gram-positive or Gram-negative bacteria are in agreement with a previous report [30]. However, this is the first investigation to demonstrate that alcohol reduces NETosis and NET-mediated extracellular bacterial killing for both Gram-negative and Gram-positive pathogens as a mechanism of suppression of innate immune responses.

Hematopoietic cells in tissues produce several neutrophil chemokines, including CXCL1 [35, 36] and CXCL2 (aka MIP-2) [37, 38] and resident cells, including epithelial and endothelial cells, produce another neutrophil chemokine CXCL5 (aka LIX) [39, 40]. The findings from our previous studies demonstrate that both hematopoietic and nonhematopoietic cell-derived CXCL1 is essential for neutrophil-dependent host defense [11, 41, 42]. Regardless of the cellular origin, we found that the inflammatory response to sepsis was dependent on CXCL1 as evidenced by neutrophil influx, bacterial clearance, and mortality [11]. We have also shown that the expression of key inflammatory cytokines and chemokines was dependent upon CXCL1 during polymicrobial sepsis. In the present study, we have demonstrated that CXCL1 rescues impaired host defense function in alcohol-challenged septic mice.

Despite the fact that CLP model is shown to be useful to understand the host defense mechanisms to microbes, several technical variations can modulate the degree of inflammation induced by CLP. The size of the needle puncture and number of punctures will regulate endpoint of polymicrobial sepsis [43]. Also, we only used a single sized needle with defined number of punctures in this investigation. We did not use antibiotics in mice since antibiotics can induce bacterial clearance and reduce bacterial dissemination and modulate the severity of sepsis [44, 45]. We used only male mice in our experiments and in general, male mice are more susceptible to CLP than are female mice [46, 47].

In conclusion, the present study furthers a number of advancements in our understanding of the biological role of acute alcohol on the host innate immune response to polymicrobial sepsis. Moreover, alcohol intoxication impairs neutrophil recruitment, NETosis, and ROS production resulting in decreased bacterial killing, in response to polymicrobial sepsis in mice. Furthermore, alcohol-induced defects in NETosis and NET-mediated killing in mice correlates with the results observed in human neutrophils. While the fact that rCXCL1 rescues the alcohol-induced phenotypes to levels of BSA treated animals after CLP suggests that rCXCL1 can compensate the alcohol-induced immunosuppression. The observation that rCXCL1 treatment induces a similar increase in alcohol-treated and -untreated animals argues in favor of independent functions of CXCL1.

Since chronic alcohol exposure also has substantial effect on the innate immune system, future studies are required to establish if chronic alcohol exposure decreases NETosis and NET-mediated bacterial killing, and whether CXCL1 rescues impaired host defense in chronic alcohol-mediated immunosuppression in septic mice. Furthermore, needs to be determined by future studies whether CXCL2 and CXL5 can rescue alcohol-induced immunosuppression in septic mice.

## Methods

### Ethics statement

All animal experiments were conducted in accordance with the recommendations in the Guide for the Care and Use of Laboratory Animals of the National Institutes of Health. The animal studies protocols were approved by the Institutional Animal Care and Use Committee at Louisiana State University Protocol numbers (16–031, 16–012). Human blood collection from adult health volunteers was approved by the Louisiana State University Institutional Review Board (LSU IRB). All donors provided informed written consent.

### Animals

C57Bl/6 male mice (8–12 weeks) and CXC ligand 1 (CXCL1) knockout male mice weighing 22 to 28 g were used in all experiments.

### Cecal ligation and puncture (CLP)

A mild form of polymicrobial sepsis (causing 50% mortality) was induced by the CLP method as previously described [48]. In brief, male mice were anesthetized and the cecum was punctured with a 21-gauge needle. Saline (1 ml) was injected subcutaneously after CLP. A small amount of fecal material was extruded through the puncture, and the cecum was repositioned into the peritoneal cavity. Sham mice were subjected to laparotomy but without CLP.

### *In vivo* alcohol challenge

Acute alcohol administration was conducted according to protocol previously established [49]. In brief, 32% ethanol at a concentration of 6 g/kg was given 30 min prior to CLP to alcohol recipient group of mice by gavage. Control mice received an equal volume of saline. In another set of experiments, 1 μg rCXCL1 or bovine serum albumin (BSA) was administrated immediately after CLP in acute alcohol- and saline-challenged mice.

### Survival studies

Survival of both groups of WT mice and CXCL1 (KC) KO mice (alcohol-given and saline-given (control) mice) that underwent CLP or sham surgery was monitored every 12 h up to 10 days and survival curves were plotted at 24 h intervals. In another set, the same procedure was followed but 1 μg of rCXCL1 or BSA was i.p. administered just before CLP.

### Peritoneal lavage

Cells from peritoneum were obtained by peritoneal lavage with 4 ml of warm heparinized RPMI 1640 medium (BRL, Bethesda, MD) and total leukocytes (WBC) were enumerated by a hemocytometer whereas neutrophil and macrophage percentages were counted manually by a light microscope after staining with Diff-Quik [11]. Peritoneal fluid was obtained by washing the peritoneum with saline (peritoneal lavage) which was centrifuged to obtain peritoneal cells (neutrophils) in the pellet. Chemokine and antimicrobial protein measurement in peritoneal fluid and serum is described in S1 Methods.

### Bacterial burden

Bacterial burdens in multiple organs were determined by enumerating bacterial numbers in CFUs as previously described [41]. The organs of CLP mice were weighed and homogenized in 1 ml of isotonic saline using a homogenizer. The tissues were allowed to sediment at room temperature for 10 min, and the supernatants were collected. The tissue samples and peritoneal lavage were serially diluted and aliquots (20 μl) of each sample were plated on tryptic soy agar plates. The number of colonies was counted following incubation at 37 °C overnight.

### Western blotting

At the designated times, peritoneal cells (neutrophils) were harvested and lysed with cell lysis buffer containing 0.1% Triton X-100 and complete protease and phosphatase inhibitor cocktail as described [42]. Equal amounts of proteins were separated by SDS-PAGE, and transferred to a polyvinyl difluoride membrane. The nonspecific binding sites on the membrane were blocked with 5% non-fat dry milk in TBS supplemented with 0.1% Tween 20 for 1 h before proteins were allowed to react with specific primary antibodies against peptidylarginine deiminase-4 (PAD-4) (Cell Signaling), neutrophil elastase (Abcam), Gp91^phox^ (Abcam), P47^phox^ (Santa Cruz), P67^phox^ (Santa Cruz) and GAPDH (Cell Signaling) at 4°C overnight. The membrane was washed three times with TBS containing 0.1% Tween 20 (0.1% TBST) and incubated with horseradish peroxidase-conjugated secondary antibody for 1 h at room temperature. The immunoreactive bands in the membrane were detected by the chemiluminescence method (Amersham). Percent of total area was calculated for each band for PAD-4 using NIH ImageJ 1.51d software, and expressed relative to neutrophil elastase bands as the mean ± SE of three bands per group.

### Purification of neutrophils

Neutrophils were purified from the bone marrow cells by negative magnetic selection using the kit from StemCell Technologies (Vancouver, BC, Canada) as described in our earlier publication [42]. Briefly, neutrophils were purified from bone marrow cells using a custom mixture containing multiple Abs (anti-CD5, -CD4, -CD45R/B220, -TER119, -F4/80, CD11c, and -c-Kit). First, bone marrow cells were incubated in RoboSep buffer at 4°C containing Ab mixture. Bone marrow cells were then placed in the EasySep magnet. Purified neutrophils samples were poured off into the new tube, washed, and resuspended in RPMI 1640 containing 10% FBS. Purified cells were then counted using a hemocytometer and used for experiments. Neutrophils were purified from peripheral blood of healthy donors using a negative magnetic selection kit from StemCell Technologies (Vancouver, British Columbia, Canada) as described in an earlier publication [17].

### Kinetics of NETosis

NETs were quantified using fluorometry as described in previous publications [11, 14]. In brief, mouse peritoneal cells (neutrophils) from peritoneal lavage at 24 h post-CLP or *E*. *coli*- or *S*. *aureus*-infected mouse bone marrow-derived neutrophils were cultured in 96-well plates, added SYTOX Green and monitored for NETosis at every h up to 8 h. A non-cell-permeant DNA binding dye, SYTOX Green, was added to the neutrophils at a concentration of 5 μM to assess extracellular DNA as a measurement of NETosis. The relative fluorescence units of treated cells were obtained after subtracting the fluorescence of unstimulated neutrophils. In *in vitro* experiments, purified bone marrow-derived neutrophils (1×10^5^ cells/well) were cultured in 96-well plates, added SYTOX Green and monitored NETosis at every h up to 8 h after stimulation with *E*. *coli* (MOI 1) or *S*. *aureus* (MOI 1). The relative fluorescence units of treated cells were obtained after subtracting the fluorescence of unstimulated neutrophils.

To make sure that dead cells by either apoptosis or necrosis did not contribute to higher fluorescence, cell viability was examined using the trypan blue exclusion assay. This indicated that at least 85–90% of the peritoneal cells were viable after 6 h and 24 h post-CLP. The viability of bone marrow-derived mouse and peripheral blood-derived human neutrophils was >90% up to 8 h.

### NETosis by fluorescence and electron microscopy

Immunofluorescence of the peritoneal cells (neutrophils) with CLP was performed to detect NETosis as described previously (16). Peritoneal cells (neutrophils) and *E*. *coli*- or *S*. *aureus*-infected mouse bone marrow-derived neutrophils were analyzed in a similar manner for NETosis using double-positive cells using DNA dye-SYTOX Green and H3-Cit staining. Scanning electron microscopy (16) (FEI Quanta 200, USA) was performed to examine NETosis in mouse peritoneal cells (neutrophils) and bone marrow-derived neutrophils following the protocols described in a previous publication [21]. *E*. *coli* (ATCC 25922) and *S*. *aureus* (ATCC BAA-1717) were used to stimulate neutrophils as described previously [50]. Alcohol (25 mM or 250 mM) was added to the media just prior to infection. More details about *in vitro* NET formation by immunofluorescence microscopy following bacterial challenge and PMA are described in S1 Methods. The details about transmission electron microscopy used to demonstrate granules in neutrophils are described in S1 Methods.

### ROS measurement

ROS+ peritoneal cells (neutrophils) from saline-challenged and alcohol intoxicated mice following CLP were enumerated using immunofluorescence microscopy. The level of total ROS in peritoneal cells (neutrophils) and in peritoneal fluid after induction of CLP was measured using the Fluorescent ROS Detection Kit (CellROX, Life Technologies) and the data expressed as relative fluorescent units (RFUs). For peritoneal fluid, RFUs were adjusted based on leukocyte numbers in the peritoneal lavage.

### *In vitro* alcohol treatment

Mouse bone marrow-derived neutrophils were treated with either 25 mM (low dose) or 250 mM (high dose) of ethanol in saline prior to challenge with either *E*. *coli* or *S*. *aureus* at an MOI of 1. To determine NET-mediated bacterial killing, cells were treated with DNase (100 U/well) 30 min prior to infection.

### Extracellular bacterial killing assay

A neutrophil killing assay was performed *in vitro* at different time points as reported earlier [42]. First, we treated mouse bone marrow-derived and human peripheral blood-derived neutrophils (each 0.25 X 10^5^ cells/ 0.2 ml of DMEM supplemented with 10% FBS) with cytochalasin D (10 μg/ml) for 20 min to block phagocytosis prior to infection with either *E*. *coli* or *S*. *aureus* (MOI 1). Alcohol (25 mM) was added to the media just prior to infection. These were added to the wells containing neutrophils, and the cell culture plates (96 well) were incubated at 37°C for 30 min, 0.5 h, 1 h and 1.5 h. At designated time points, the plates were centrifuged to remove neutrophils, supernatants were collected, diluted in sterile isotonic saline, plated on Tryptic Soy Agar plates, and incubated overnight at 37°C for bacterial enumeration. In another set of experiments, bone marrow-derived neutrophils were treated with 5 nM rCXCL1 or BSA for one hour and infected with either *E*. *coli* or *S*. *aureus* at an MOI of 1 in the presence of 25 mM alcohol in saline.

### Data analysis

Data presented here were from 2–3 independent experiments. Data were tested for normality using Shapiro-Wilk normality test. Data with Gaussian (normal) distribution were compared using Student’s t-test (between 2 groups) or with one-way ANOVA (>2 groups) with Tukey’s multiple comparison test. Statistical analyses were performed using GraphPad Prism (v.5.03) (GraphPad, San Diego, CA). Survival curves were compared by the Wilcoxon rank sign test. Data are expressed as mean ± SE. Differences in values were defined as significant at a *p* value of less than 0.05, 0.01 or 0.001.

## Supporting information

S1 Methods Detailed methods related to chemokine and antimicrobial peptide measurement, transmission electron microscopy and *in vitro* NET formation using immunofluorescence microscopy are described. (DOCX)

S1 Fig Reduced production of CXCL1 (KC) in peritoneal fluid of alcohol-intoxicated mice following CLP. **A-B**. Acute alcohol-treated and saline-challenged mice were subjected to sham or CLP. The concentration (in pg/mL) of CXCL1 was quantified in serum (A) and peritoneal fluid (B) at 6 and 24 h post-CLP using ELISA. (n = 5-8/group; *, p<0.05).
(TIF)

S2 Fig **A. Neutrophil morphology and the presence of granules are not different in peritoneal cells (neutrophils) between alcohol-treated and saline-challenged mice**. Transmission electron microscopy (TEM) of mouse peritoneal cells (neutrophils). These cells contain numerous types of granules in cytosol (dark colored, primary; big and light colored, secondary and small and light colored, tertiary granules) and lobulated nuclei. N, nucleus; G, granules. This image is a representative image of 3 images with identical results. TEM original magnification: 4000x (upper panel); 20000x (lower panel) **B. Attenuated production of cathelicidin in peritoneal fluid of alcohol-treated mice following CLP**. Alcohol-treated and saline-challenged mice were subjected to sham or CLP and the concentration of cathelicidin was quantified in serum and peritoneal fluid at 6 and 24 h post-CLP (in pg/ml) using ELISA. (n = 4-6/group; *, p<0.05).
(TIF)

S3 Fig Alcohol impairs NET formation in mouse bone marrow-derived neutrophils in response to *E*. *coli* infection. Mouse bone marrow-derived neutrophils treated with 25 mM alcohol exhibit diminished NET formation in response to a Gram-negative bacterial infection. Mouse neutrophils harvested from C57BL/6 mice were pretreated with 25 mM alcohol before infection with *E*. *coli*, added SYTOX Green, allowed to form NETs, and then fixed after 8 h. Neutrophils were stained with citrullinated H3 (H3Cit) Ab to observe citrullinated histones and DAPI to visualize intracellular DNA in fixed cells. Double-positive cells are indicated by arrowheads to demonstrate NET formation. DAPI colocalizes with SYTOX Green and H3Cit. This image is a representative of 20 random images from 3 independent experiments; (n = 4-6/group; *, p<0.05) NET forming neutrophils are indicated by the arrows on merged images and original magnification 20x.
(TIF)

S4 Fig Alcohol decreases NET formation in mouse bone marrow-derived neutrophils triggered by phorbol 12-myristate 13-acetate (PMA). **A**, Mouse bone marrow-derived neutrophils treated with 25 or 250 mM alcohol exhibit diminished NET formation in response to a non-infectious stimulus (PMA). Mouse neutrophils harvested from C57BL/6 mice were pretreated with either 25 or 250 mM alcohol before stimulation with PMA. SYTOX Green was added and allowed to form NETs, and then fixed 8 h post-stimulation. Neutrophils were stained with citrullinated H3 Ab to visualize citrullinated histones after the cells were fixed. Double-positive cells are indicated by arrowheads as evidence of NET formation. **B**, Twenty random images were selected from one experiment and quantified for the presence of NET-positive neutrophils from 3 independent experiments. (n = 4-6/group; *, p<0.05); NET forming neutrophils are indicated by the arrows on merged images and original magnification 20x.
(TIF)

S5 Fig Alcohol reduces NET formation and NET-mediated bacterial killing in human peripheral blood-derived neutrophils in response to *E*. *coli* infection. **A**, Human neutrophils treated with either 25 or 250 mM alcohol exhibit diminished NET formation in response to a Gram-negative (*E*. *coli*) bacterial infection. Human neutrophils purified from peripheral blood were pretreated with either 25 or 250 mM alcohol before infection with *E*. *coli*. SYTOX Green was added to neutrophils and allowed to undergo NET formation, and then fixed 8 h post-infection. Neutrophils were stained with citrullinated H3 Ab to visualize citrullinated DNA. Double-positive cells are indicated by arrowheads as evidence of NET formation. Images presented are representative of three independent experiments (each in duplicate). **B**, A total of 20 random images were selected from three experiments and quantified for the presence of NET-positive neutrophils. (*, p<0.05). **C**, Kinetic analysis of NET formation by *E*.*coli*-infected human neutrophils treated with alcohol. Mouse neutrophils were pretreated with either 25 mM or 250 mM alcohol before infection with *E*. *coli* and observed for SYTOX Green DNA stain every hour over a period of 8 h using fluorimetry as Relative fluorescent unit (RFU) to determine NET formation (*, p<0.05). **D**, Evaluation of NET formation by SEM. Human peripheral blood neutrophils were pretreated with either 25 or 250 mM alcohol before infection with *E*. *coli* and incubated for 8 h to observe NET formation by SEM. **E**, Alcohol-treated human neutrophils exhibited diminished NET-mediated killing activity. Bacterial killing capacity of *E*. *coli*-infected, alcohol-treated and untreated human neutrophils was determined by assessing extracellular (CFUs) at 8h post-infection with *E*. *coli* (MOI 1) in the presence or absence of DNase (100 U/well). A total of four to five donors/group were used. (*, p<0.05); NET forming neutrophils are indicated by the arrows on merged images and original magnification 20x. SEM magnification 8000x.
(TIF)

S6 Fig Alcohol attenuates NET formation and NET-mediated killing by human neutrophils triggered by *S*. *aureus* infection. **A**, Human neutrophils treated with 25 or 250 mM alcohol exhibited diminished NET formation in response to *S*. *aureus* infection. Human neutrophils purified from the blood of healthy donors were pretreated with either 25 or 250 mM alcohol prior to infection with *S*. *aureus*. SYTOX Green was added to neutrophils and allowed to form NETs up to 8 h, and were fixed with 4% formaldehyde. Neutrophils were then stained with citrullinated histone H3 Ab to visualize citrullinated DNA. Double-positive cells are indicated by arrowheads as evidence of NET formation Images presented are representative of three independent experiments (each in duplicate). **B**, A total of 20 random images were selected from three experiments and the presence of double (NET)-positive neutrophils was quantified. (*, p<0.05). **C**, Kinetic analysis of NET formation by human neutrophils, pretreated with either 25 or 250 mM alcohol before infection with *S*. *aureus* and monitored with SYTOX Green up to 8 h. Relative fluorescent intensity was determined to evaluate NET formation (*, p<0.05). **D**, Evaluation of NET formation in human neutrophils by SEM. Neutrophils were pretreated with either 25 mM or 250 mM alcohol before infection with *S*. *aureus* and incubated for 8 h to allow for NET formation. NET formation by neutrophils was analyzed by SEM. **E**, Alcohol-treated human neutrophils displayed diminished NET-mediated killing activity. Bacterial killing capacity of *S*. *aureus*-infected, alcohol-treated and untreated human neutrophils was determined at 8 h post-infection. Experiments were carried out independently, three times, each in duplicate. A total of four to five donors/group were used. *, p<0.05; NET forming neutrophils are indicated by the arrows on merged images and original magnification 20x. SEM magnification 8000x.
(TIF)
