# Supplementary material for: Diminished neutrophil extracellular trap (NET) formation is a novel innate immune deficiency induced by acute ethanol exposure in polymicrobial sepsis, which can be rescued by CXCL1
Source: PLoS Pathog. 2017 Sep 18;13(9):e1006637. doi: 10.1371/journal.ppat.1006637 (PMC5626520; doi:10.1371/journal.ppat.1006637)
Supplement: S1 Methods — (DOCX) [file ppat.1006637.s001.docx]

**S1 Methods**

**Chemokine and antimicrobial peptide measurement.** Mouse CXCL1 (KC) and cathelicidin levels in serum and/or peritoneal lavage fluid were measured by a double-ligand enzyme-linked immunosorbent assay (ELISA) as described in previous publications [1, 2].

**Transmission Electron Microscopy.** Neutrophils were prepared for transmission electron microscopy (TEM) as described earlier [3]. Briefly, cells were fixed with 2% formaldehyde, 2% glutaraldehyde, 0.1M sodium cacodylate trihydrate, 5 mM CaCl_2_ and 2.5% sucrose at pH 7.4 buffer for 3 h at room temperature. Cells were washed thrice with ice-cold 0.1M sodium cacodylate buffer supplemented with 2.5% sucrose and incubated with Palade’s OsO_4_ supplemented with 1% osmium tetroxide in acetate/veronal solution for 1 h. Cells were then rinsed with doubled distilled water, the neutrophils were dehydrated at increasing ethanol concentrations, such as 50, 70%, 95, and 100% for 15 min. The samples were then allowed to polymerize in 100% Epon blocks for 48 h at 80°C. Cell sections were stained with uranyl acetate and lead citrate and observed in a JEOL JEM-1011 transmission electron microscope (JEOL, Inc., Peabody, MA).

***In vitro* alcohol treatment to determine NET formation.** Mouse bone marrow-derived neutrophils or human peripheral blood-derived neutrophils were treated with either 25 mM (low dose) or 250 mM (high dose) of ethanol prior to challenge with either *E. coli* or *S. aureus* at a multiplicity of infection (MOI) of 1.0. In additional experiments, neutrophils were stimulated with Phorbol 12-myristate 13*-*acetate (PMA) at a concentration of 100 nM instead of infection.

**Immunofluorescence Microscopy.** Immunofluorescence of the cells was performed to detect NET formation as described previously [4]. Peritoneal cells (neutrophils) and *E. coli*-infected mouse bone marrow-derived neutrophils were analyzed in a similar manner for NET formation using double positive cells using DNA dye (SYTOX Green) and H3-Cit staining. DAPI was used to show that both double- (SYTOX Green and H3Cit) positive stained cells are also DAPI-positive both in the presence and absence of alcohol.

**References**

1. Cai S, Batra S, Del Piero F, Jeyaseelan S. NLRP12 modulates host defense through IL-17A-CXCL1 axis. Mucosal Immunol. 2016;9(2):503-14. doi: 10.1038/mi.2015.80. PubMed PMID: 26349659.

2. Cai S, Batra S, Langohr I, Iwakura Y, Jeyaseelan S. IFN-gamma induction by neutrophil-derived IL-17A homodimer augments pulmonary antibacterial defense. Mucosal Immunol. 2015. doi: 10.1038/mi.2015.95. PubMed PMID: 26349661; PubMed Central PMCID: PMCPMC4785101.

3. Timmer AM, Timmer JC, Pence MA, Hsu LC, Ghochani M, Frey TG, et al. Streptolysin O promotes group A Streptococcus immune evasion by accelerated macrophage apoptosis. J Biol Chem. 2009;284(2):862-71. doi: 10.1074/jbc.M804632200. PubMed PMID: 19001420; PubMed Central PMCID: PMCPMC2613605.

4. Jin L, Batra S, Douda DN, Palaniyar N, Jeyaseelan S. CXCL1 contributes to host defense in polymicrobial sepsis via modulating T cell and neutrophil functions. J Immunol. 2014;193(7):3549-58. doi: 10.4049/jimmunol.1401138. PubMed PMID: 25172493; PubMed Central PMCID: PMCPMC4170008.
