# Supplementary figures and images for: Diminished neutrophil extracellular trap (NET) formation is a novel innate immune deficiency induced by acute ethanol exposure in polymicrobial sepsis, which can be rescued by CXCL1
Source: PLoS Pathog. 2017 Sep 18;13(9):e1006637. doi: 10.1371/journal.ppat.1006637 (PMC5626520; doi:10.1371/journal.ppat.1006637)

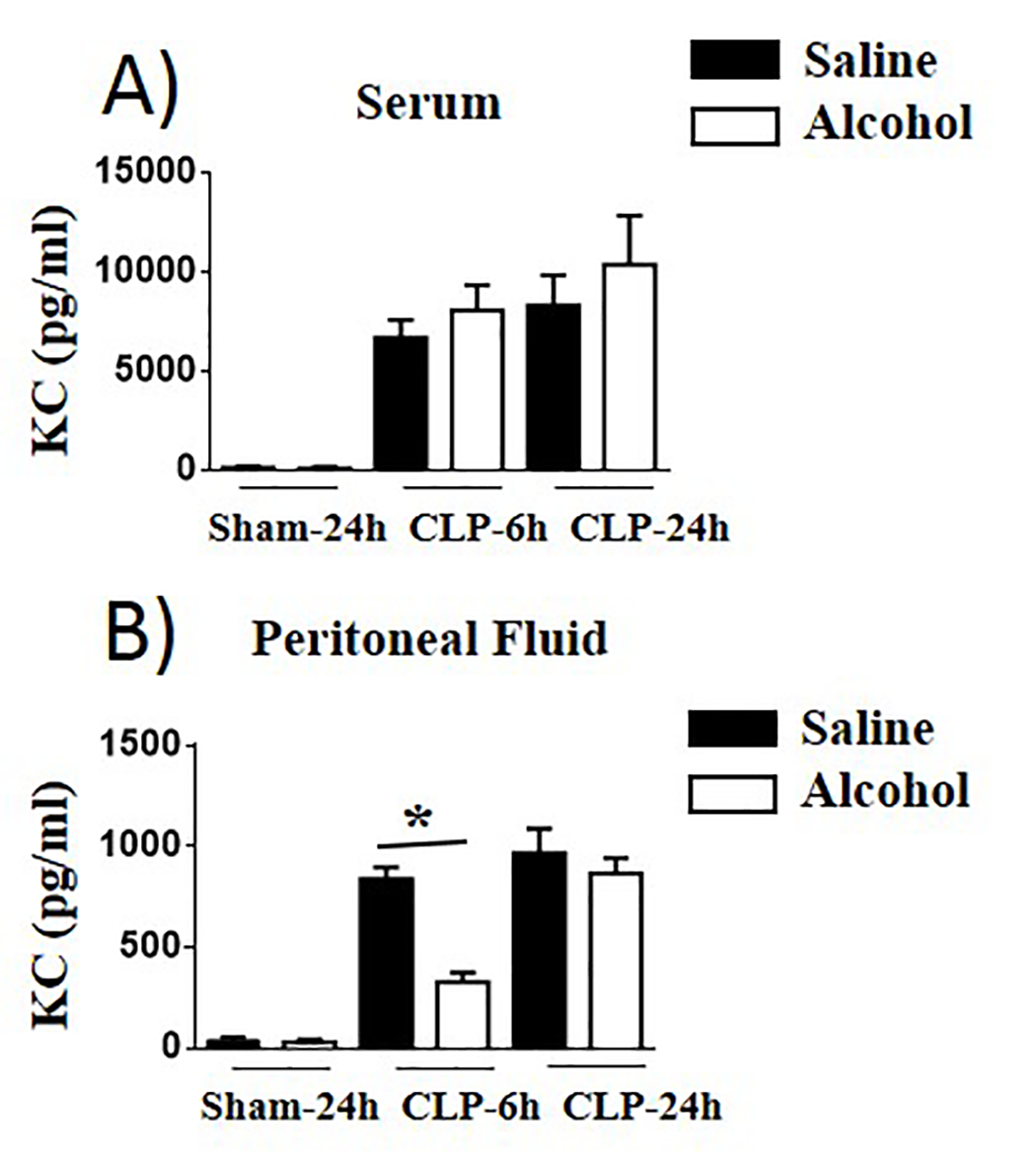

Supplement: S1 Fig — A-B. Acute alcohol-treated and saline-challenged mice were subjected to sham or CLP. The concentration (in pg/mL) of CXCL1 was quantified in serum (A) and peritoneal fluid (B) at 6 and 24 h post-CLP using ELISA. (n = 5-8/group; *, p<0.05). (TIF) [file ppat.1006637.s002.tif]

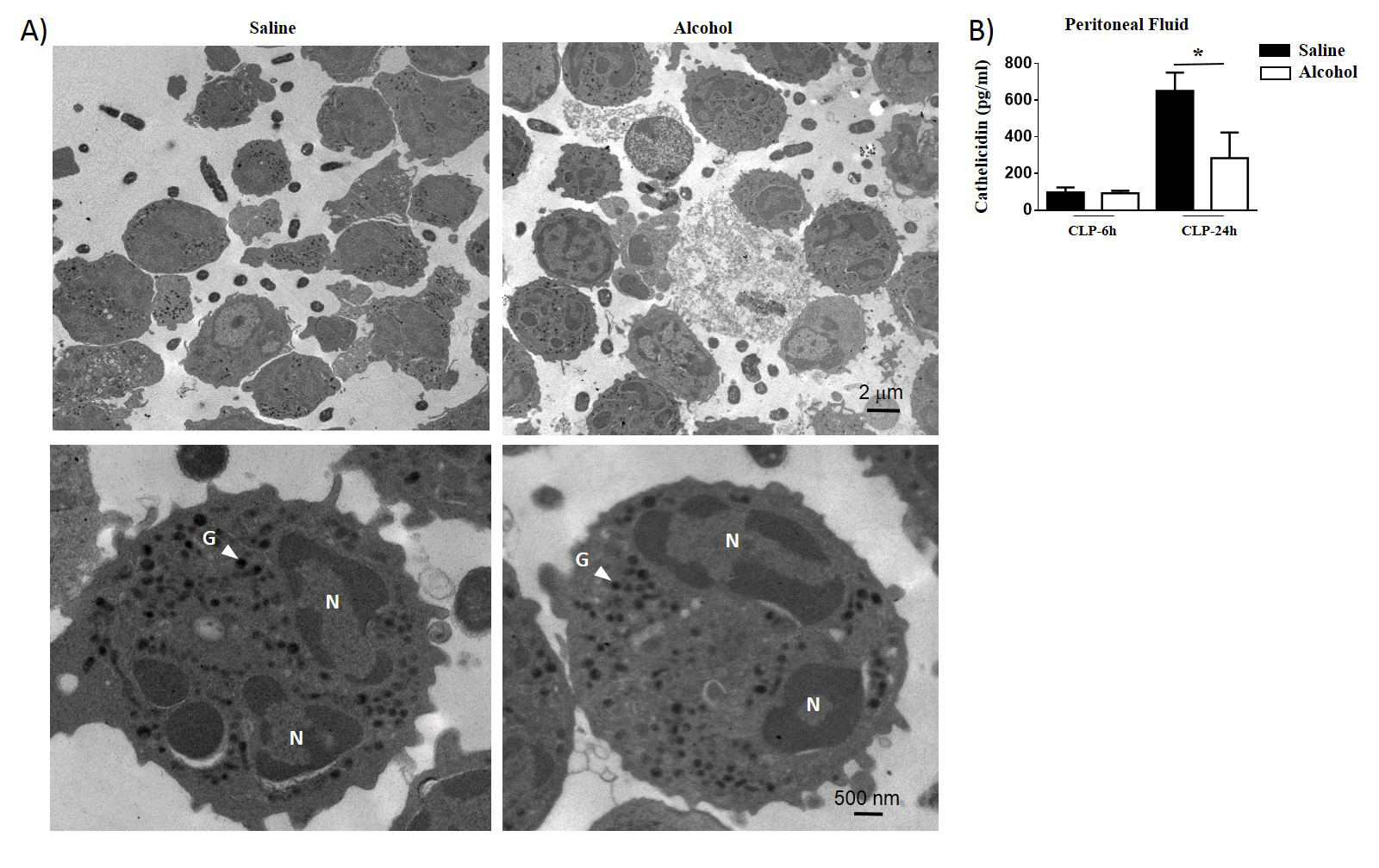

Supplement: S2 Fig — A. Neutrophil morphology and the presence of granules are not different in peritoneal cells (neutrophils) between alcohol-treated and saline-challenged mice. Transmission electron microscopy (TEM) of mouse peritoneal cells (neutrophils). These cells contain numerous types of granules in cytosol (dark colored, primary; big and light colored, secondary and small and light colored, tertiary granules) and lobulated nuclei. N, nucleus; G, granules. This image is a representative image of 3 images with identical results. TEM original magnification: 4000x (upper panel); 20000x (lower panel) B. Attenuated production of cathelicidin in peritoneal fluid of alcohol-treated mice following CLP. Alcohol-treated and saline-challenged mice were subjected to sham or CLP and the concentration of cathelicidin was quantified in serum and peritoneal fluid at 6 and 24 h post-CLP (in pg/ml) using ELISA. (n = 4-6/group; *, p<0.05). (TIF) [file ppat.1006637.s003.tif]

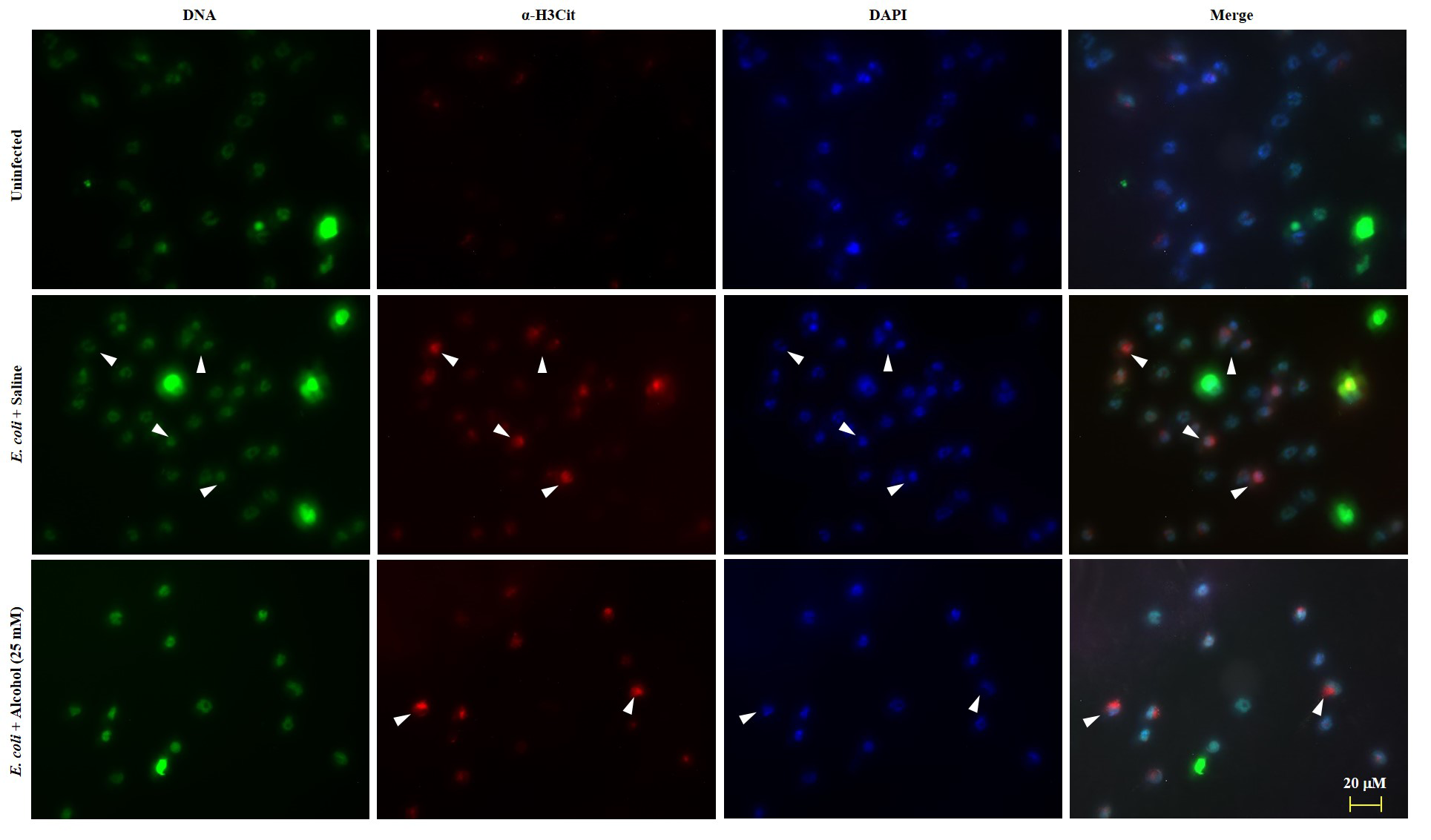

Supplement: S3 Fig — Mouse bone marrow-derived neutrophils treated with 25 mM alcohol exhibit diminished NET formation in response to a Gram-negative bacterial infection. Mouse neutrophils harvested from C57BL/6 mice were pretreated with 25 mM alcohol before infection with E. coli, added SYTOX Green, allowed to form NETs, and then fixed after 8 h. Neutrophils were stained with citrullinated H3 (H3Cit) Ab to observe citrullinated histones and DAPI to visualize intracellular DNA in fixed cells. Double-positive cells are indicated by arrowheads to demonstrate NET formation. DAPI colocalizes with SYTOX Green and H3Cit. This image is a representative of 20 random images from 3 independent experiments; (n = 4-6/group; *, p<0.05) NET forming neutrophils are indicated by the arrows on merged images and original magnification 20x. (TIF) [file ppat.1006637.s004.tif]

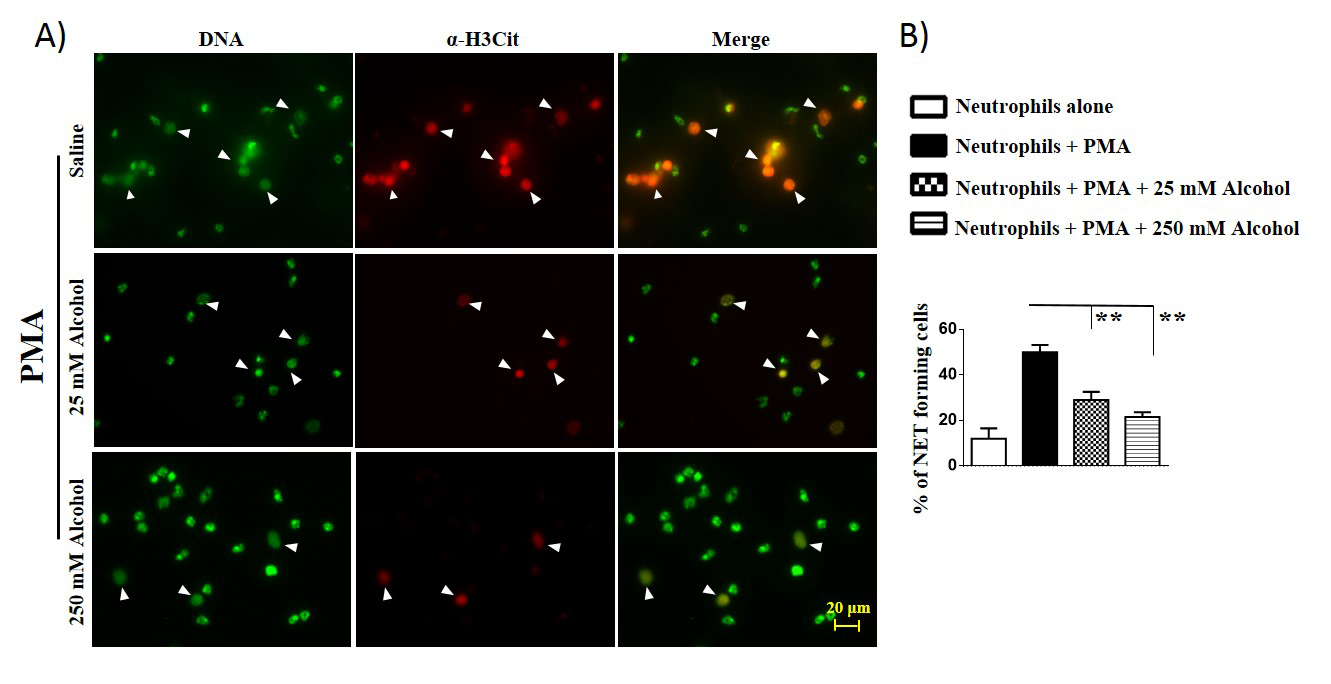

Supplement: S4 Fig — A, Mouse bone marrow-derived neutrophils treated with 25 or 250 mM alcohol exhibit diminished NET formation in response to a non-infectious stimulus (PMA). Mouse neutrophils harvested from C57BL/6 mice were pretreated with either 25 or 250 mM alcohol before stimulation with PMA. SYTOX Green was added and allowed to form NETs, and then fixed 8 h post-stimulation. Neutrophils were stained with citrullinated H3 Ab to visualize citrullinated histones after the cells were fixed. Double-positive cells are indicated by arrowheads as evidence of NET formation. B, Twenty random images were selected from one experiment and quantified for the presence of NET-positive neutrophils from 3 independent experiments. (n = 4-6/group; *, p<0.05); NET forming neutrophils are indicated by the arrows on merged images and original magnification 20x. (TIF) [file ppat.1006637.s005.tif]

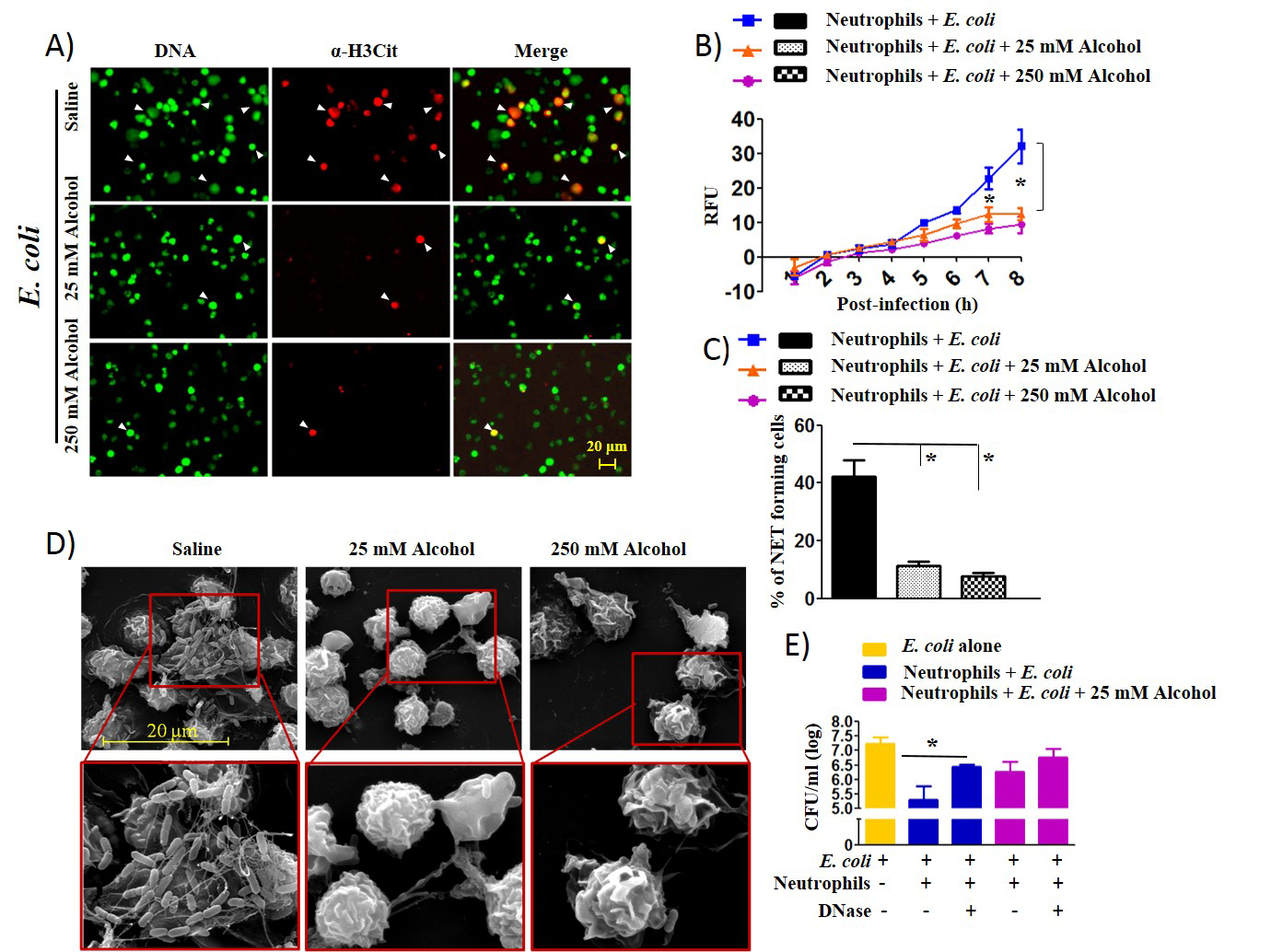

Supplement: S5 Fig — A, Human neutrophils treated with either 25 or 250 mM alcohol exhibit diminished NET formation in response to a Gram-negative (E. coli) bacterial infection. Human neutrophils purified from peripheral blood were pretreated with either 25 or 250 mM alcohol before infection with E. coli. SYTOX Green was added to neutrophils and allowed to undergo NET formation, and then fixed 8 h post-infection. Neutrophils were stained with citrullinated H3 Ab to visualize citrullinated DNA. Double-positive cells are indicated by arrowheads as evidence of NET formation. Images presented are representative of three independent experiments (each in duplicate). B, A total of 20 random images were selected from three experiments and quantified for the presence of NET-positive neutrophils. (*, p<0.05). C, Kinetic analysis of NET formation by E.coli-infected human neutrophils treated with alcohol. Mouse neutrophils were pretreated with either 25 mM or 250 mM alcohol before infection with E. coli and observed for SYTOX Green DNA stain every hour over a period of 8 h using fluorimetry as Relative fluorescent unit (RFU) to determine NET formation (*, p<0.05). D, Evaluation of NET formation by SEM. Human peripheral blood neutrophils were pretreated with either 25 or 250 mM alcohol before infection with E. coli and incubated for 8 h to observe NET formation by SEM. E, Alcohol-treated human neutrophils exhibited diminished NET-mediated killing activity. Bacterial killing capacity of E. coli-infected, alcohol-treated and untreated human neutrophils was determined by assessing extracellular (CFUs) at 8h post-infection with E. coli (MOI 1) in the presence or absence of DNase (100 U/well). A total of four to five donors/group were used. (*, p<0.05); NET forming neutrophils are indicated by the arrows on merged images and original magnification 20x. SEM magnification 8000x. (TIF) [file ppat.1006637.s006.tif]

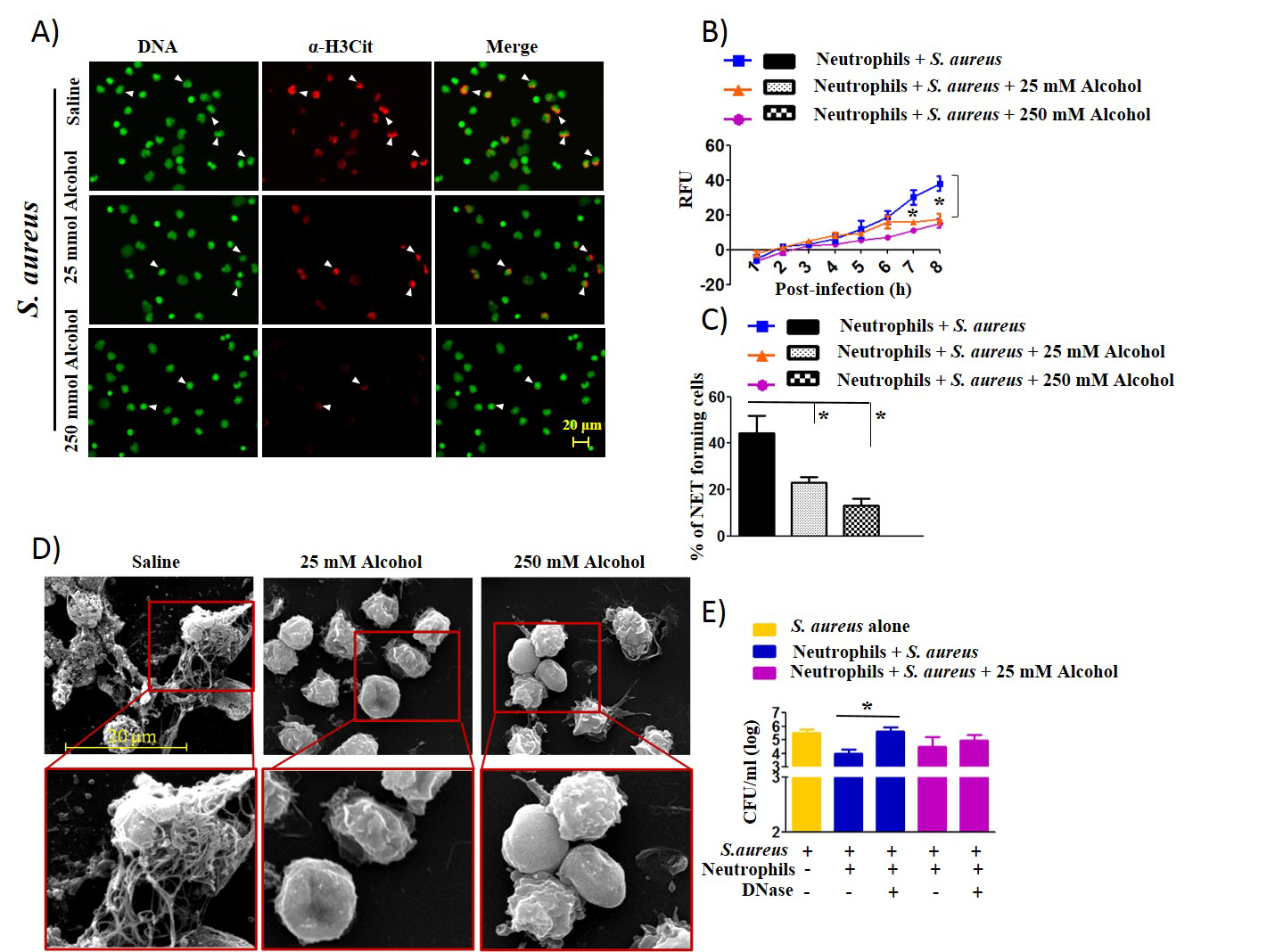

Supplement: S6 Fig — A, Human neutrophils treated with 25 or 250 mM alcohol exhibited diminished NET formation in response to S. aureus infection. Human neutrophils purified from the blood of healthy donors were pretreated with either 25 or 250 mM alcohol prior to infection with S. aureus. SYTOX Green was added to neutrophils and allowed to form NETs up to 8 h, and were fixed with 4% formaldehyde. Neutrophils were then stained with citrullinated histone H3 Ab to visualize citrullinated DNA. Double-positive cells are indicated by arrowheads as evidence of NET formation Images presented are representative of three independent experiments (each in duplicate). B, A total of 20 random images were selected from three experiments and the presence of double (NET)-positive neutrophils was quantified. (*, p<0.05). C, Kinetic analysis of NET formation by human neutrophils, pretreated with either 25 or 250 mM alcohol before infection with S. aureus and monitored with SYTOX Green up to 8 h. Relative fluorescent intensity was determined to evaluate NET formation (*, p<0.05). D, Evaluation of NET formation in human neutrophils by SEM. Neutrophils were pretreated with either 25 mM or 250 mM alcohol before infection with S. aureus and incubated for 8 h to allow for NET formation. NET formation by neutrophils was analyzed by SEM. E, Alcohol-treated human neutrophils displayed diminished NET-mediated killing activity. Bacterial killing capacity of S. aureus-infected, alcohol-treated and untreated human neutrophils was determined at 8 h post-infection. Experiments were carried out independently, three times, each in duplicate. A total of four to five donors/group were used. *, p<0.05; NET forming neutrophils are indicated by the arrows on merged images and original magnification 20x. SEM magnification 8000x. (TIF) [file ppat.1006637.s007.tif]
